# Aspirin activates resolution pathways to reprogram T cell and macrophage responses in colitis-associated colorectal cancer

**DOI:** 10.1126/sciadv.abl5420

**Published:** 2022-02-02

**Authors:** Roberta De Matteis, Magdalena B. Flak, Maria Gonzalez-Nunez, Shani Austin-Williams, Francesco Palmas, Romain A. Colas, Jesmond Dalli

**Affiliations:** 1 William Harvey Research Institute, Barts and The London School of Medicine and Dentistry, Queen Mary University of London, Charterhouse Square, London EC1M 6BQ, UK.; 2 Centre for Experimental Medicine and Rheumatology, William Harvey Research Institute, Barts and The London School of Medicine and Dentistry, Queen Mary University of London, London, UK.; 3 Centre for Inflammation and Therapeutic Innovation, Queen Mary University of London, London, UK.

## Abstract

Inflammation is linked with carcinogenesis in many types of cancer including colorectal cancer (CRC). Aspirin is recommended for the prevention of CRC, although the mechanism(s) mediating its immunomodulatory actions remain incompletely understood. Here, we demonstrate that aspirin increased concentrations of the immune-regulatory aspirin-triggered specialized proresolving mediators (AT-SPMs), including AT-lipoxin A_4_ and AT-resolvin D1, in colonic tissues during inflammation-associated CRC (I-CRC). Aspirin also down-regulated the expression of the checkpoint protein programmed cell death protein-1 in macrophages and CD8^+^ T cells from the colonic mucosa. Inhibition of AT-SPM biosynthesis or knockout of the AT-SPM receptor *Alx/Fpr2* reversed the immunomodulatory actions of aspirin on macrophages and CD8^+^ T cells and abrogated its protective effects during I-CRC. Furthermore, treatment of mice with AT-SPM recapitulated the immune-directed actions of aspirin during I-CRC. Together, these findings elucidate a central role for AT-SPM in mediating the immune-directed actions of aspirin in regulating I-CRC progression.

## Teaser

AT-SPMs mediate the immune-directed actions of aspirin in colitis-associated colorectal cancer.

## INTRODUCTION

Colorectal cancer (CRC) remains the second most common cause of cancer deaths in developed countries, despite important recent advances in treatment and detection strategies, with more than 1 million cases being diagnosed each year worldwide (*1*). Of these, only about 20% are associated with hereditary genetic traits, while most cases are associated with other factors, including chronic intestinal inflammation. The role of inflammation in CRC is supported by both experimental and clinical data demonstrating a beneficial effect of anti-inflammatory drugs, including aspirin, in preventing or delaying the development of CRC [reviewed in (*2*, *3*)].

Aspirin acetylates cyclo-oxygenase (COX) enzymes inhibiting the production of prostaglandins (PGs) and thromboxane (TX) that, in experimental settings, have been suggested to contribute to both tumor development and metastasis in CRC (*2*, *4*, *5*). Whereby, for example, prostaglandin E_2_ (PGE_2_) was recently found to promote the growth of transplantable tumors, including that of a colorectal cell line–derived tumor, via the suppression of host immunity in mice (*5*), and Thromboxane (TX)A_2_ facilitated tumor metastasis in a platelet-dependent manner in a metastasis implantation model (*4*). These findings are also supported by clinical observations where increased PGE_2_ production is observed in patients with colorectal neoplasia and is linked with colorectal carcinogenesis (*6*). Nevertheless, it is now increasingly clear that this mechanism alone does not account for the immunoregulatory actions of aspirin in regulating CRC. While acetylation of COX-1 inhibits enzyme activity, acetylated COX-2 remains enzymatically active switching its catalytic activity to the production of protective molecules termed as aspirin-triggered specialized proresolving mediators (AT-SPMs) (*7*, *8*). These include AT–lipoxin (LX) A_4_, which is produced via the stereoselective conversion of arachidonic acid (*8*), and AT–resolvin (Rv) D1 enzymatically produced from the omega-3 essential fatty acid docosahexaenoic acid (*7*). AT-SPMs actively promote the termination of inflammation by counter-regulating the production of proinflammatory mediators as well as reprograming the host immune response, actions that are mediated via the activation of cognate receptors (*9*).

Recent findings suggest that regular aspirin intake was more effective in reducing risk of CRC in those cancers that expressed higher COX-2 levels, when compared with those displaying weak or no COX-2 expression (*10*). Therefore, herein, we investigated the role of AT-SPM in the immunomodulatory actions of aspirin in inflammation-associated CRC (I-CRC).

## RESULTS

### Aspirin is protective in murine I-CRC

**Fig. 1.**
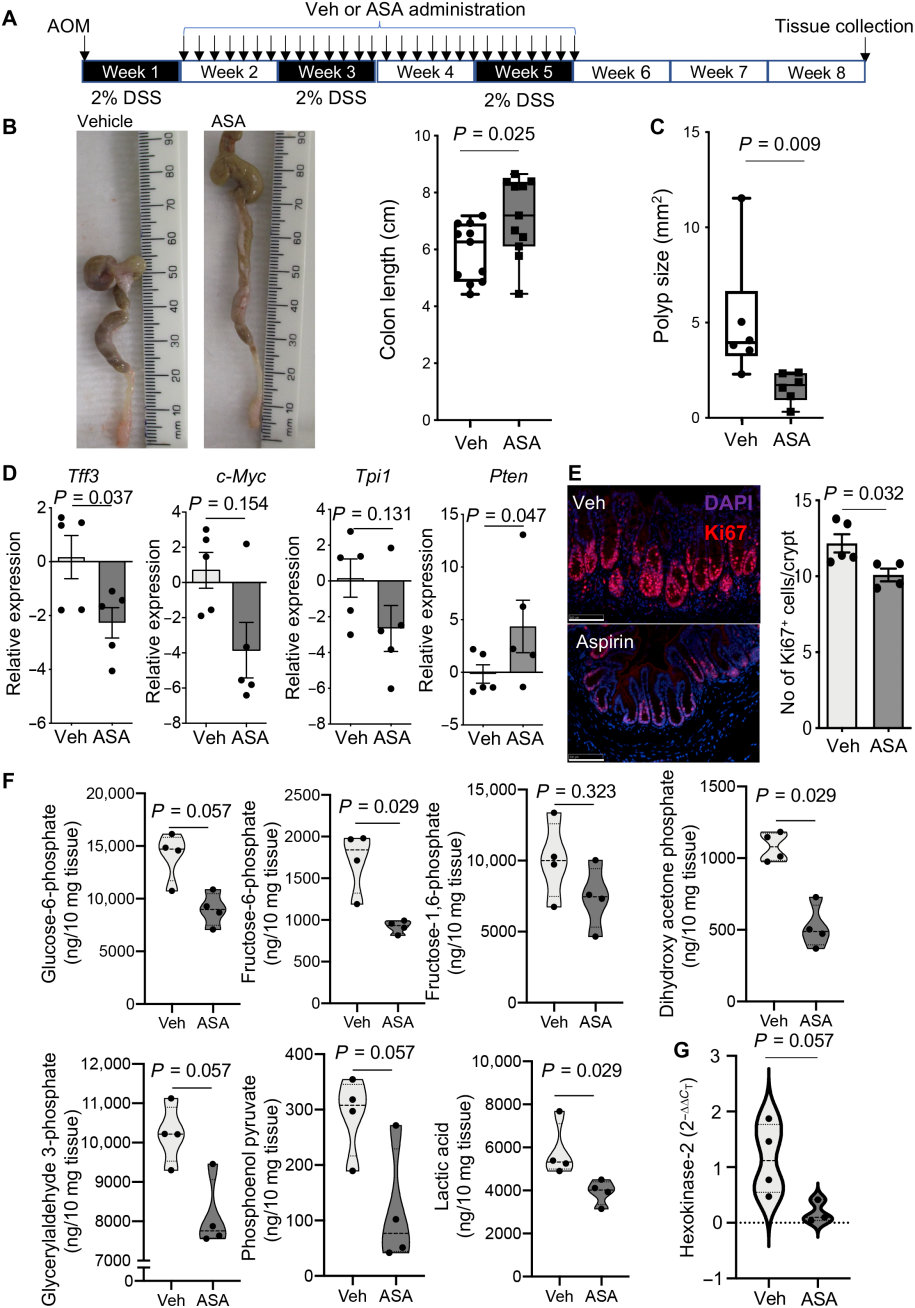
Aspirin protects against I-CRC, down-regulating the expression of tumorigenic genes and reducing tissue glycolysis. I-CRC was initiated by administrating AOM (7.5 mg/kg via intraperitoneal injection) and DSS (2% in the drinking water). After 7 days, mice were treated with 43 mg/kg of aspirin (ASA) or vehicle (Veh) via oral gavage, daily for a 4-week period. Three weeks after the last dose of aspirin, mice were culled and colons were harvested. (**A**) Experimental timeline. (**B**) Representative images (left), colon length (right), and (**C**) polyp area distal colon. Results present median together with minimum to maximum values. *n* = 11 for (B) and 6 for (C) from two distinct experiments. Photo credit: Roberta De Matteis and Shani Austin-Williams, QMUL. (**D**) Expression of *Tff3*, *c-Myc*, *Tpi1*, and *Pten* was determined in colon tissues using quantitative real-time polymerase chain reaction (qRT-PCR). (**E**) Expression of Ki67 was determined using immunofluorescence staining. Representative images (left). Results are means ± SEM. *n* = 4 to 7 mice per group from two distinct experiments. Scale bars, 100 μM. DAPI, 4′,6-diamidino-2-phenylindole. (**F**) Concentrations of metabolites in the glycolysis pathway were determined using LC-MS/MS. (**G**) Expression of hexokinase-2 (HK2) was determined using qRT-PCR in colon tissues. Results present median and quartiles. *n* = 3 to 4 mice per group. Statistical differences for (B) to (G) were evaluated using Mann-Whitney test.

To establish the mechanism by which aspirin exerts its immunomodulatory actions, we used the azoxymethane/dextran sodium sulfate (AOM/DSS) model. This model recapitulates key aspects of the histological, pathological, and molecular features of I-CRC (*11*) and is associated with enhanced COX-2 expression (*12*). We first assessed the dose-response relationships for aspirin in reducing inflammation in I-CRC, given that unremitting inflammation is the key trigger in I-CRC (*13*). Mice were treated with doses that were equivalent, using a body surface area dose conversion method (*4*), to the human low (10 mg/kg in mice = 81 mg/day in humans), intermediate (43 mg/kg in mice = 325 mg/day in humans), and high (86 mg/kg in mice = 650 mg/day in humans) doses of aspirin. Administration of aspirin 7 days after AOM/DSS initiation reduced disease severity as measured by an increase in colon length. Notably, here, we found that the intermediate dose of aspirin conferred the greatest degree of protection (fig. S1). This observation is in line with published studies demonstrating that, in mice, the intermediate equivalent dose displays similar pharmacological properties to the low dose in humans (*4*). Having observed that the 43 mg/kg dose was effective at limiting inflammation, we next investigated whether this dose also protected from carcinogenesis. For this purpose, I-CRC was initiated, and mice were treated with 43 mg/kg of aspirin as described above. Here, we found that in addition to protection against colon shortening, as previously observed, aspirin also significantly reduced polyp area (Fig. 1, A to C).

To further evaluate the molecular mechanisms regulated by aspirin in protecting against I-CRC, we explored the expression of genes linked with carcinogenesis. Here, we found that expression of the proto-oncogene C-myc, together with that of trefoil factor 3 (*Tff3*) and triosephosphate isomerase 1 (*Tpi1*), which is involved in cellular glycolysis, was down-regulated in colorectal tissue from aspirin-treated mice when compared with mice given vehicle (Fig. 1D). Furthermore, immunohistochemical analysis of colon tissues from aspirin-treated mice demonstrated a significant reduction in the number of Ki67-positive cells (Fig. 1E). These findings were coupled with a significant up-regulation of the tumor suppressor gene Phosphatase and TENsin homolog (*Pten*) (Fig. 1D). Together, these findings identify molecular targets regulated by aspirin in mice with I-CRC.

### Aspirin administration regulates colon tissue metabolism during I-CRC

It is now well appreciated that, during carcinogenesis, including I-CRC, there is a shift in tissue metabolism toward glycolysis that, in turn, leads to an alteration of host immune responses (*14*, *15*). Accordingly, we investigated the concentrations of products within the glycolytic pathway using liquid chromatography–tandem mass spectrometry (LC-MS/MS). This demonstrated a marked down-regulation in all the intermediates measured within this pathway, reaching statistical significance for most of the products, including dihydroxy acetone phosphate and lactic acid, in colonic tissues from aspirin-treated animals (Fig. 1F). This decrease in glycolytic products was coupled with a significant down-regulation in the expression of hexokinase 2, one of the rate-limiting enzymes in the glycolytic pathway (Fig. 1G).

### Regulation of macrophage phenotype and function by aspirin in I-CRC

**Fig. 2.**
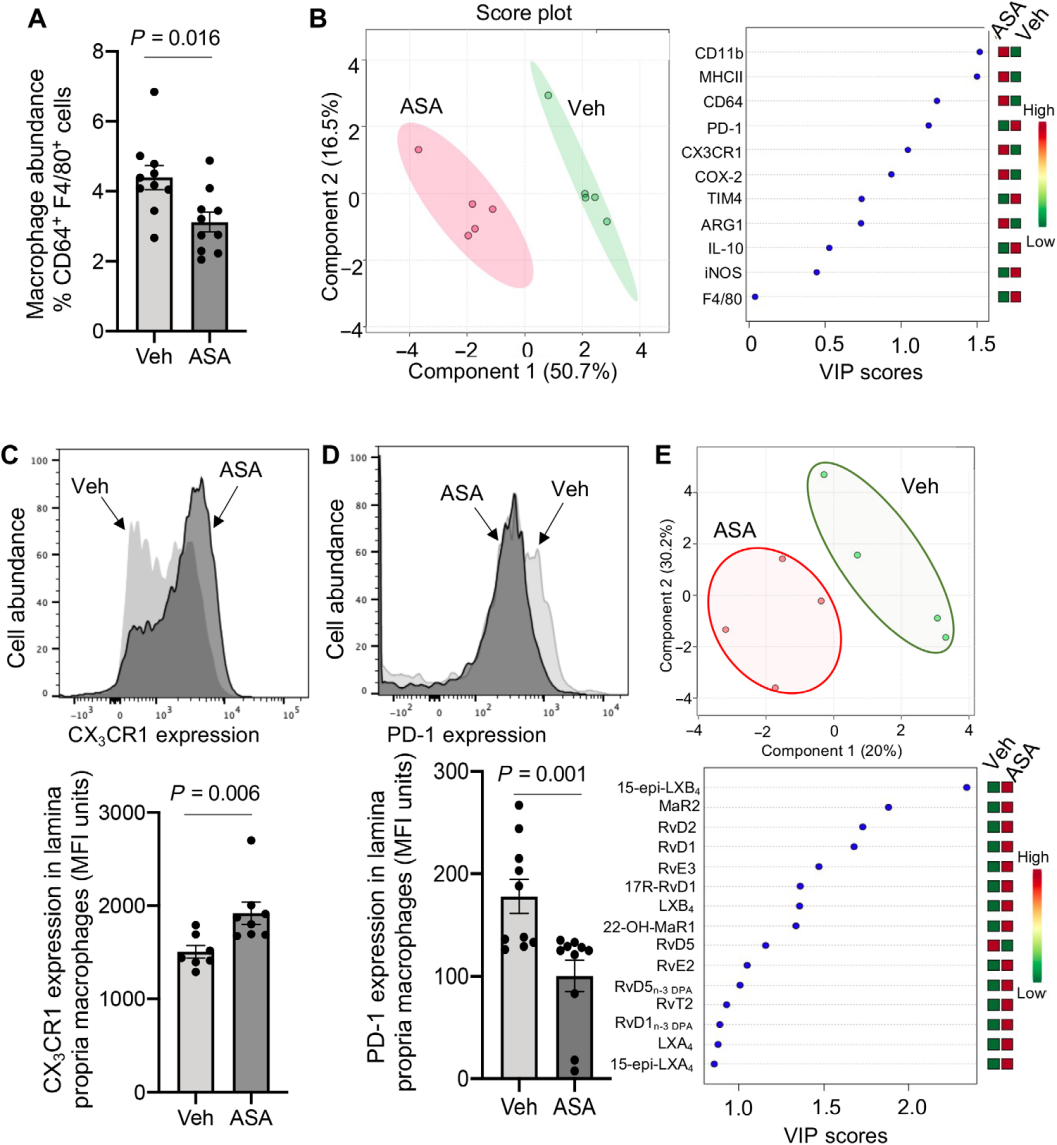
Aspirin decreases PD-1 expression and regulates macrophage trafficking and phenotype during I-CRC. I-CRC was initiated, and mice were treated as in Fig. 1. Colons were harvested, and flow cytometry was used to (**A**) measure the abundance of CD64^+^F4/80^+^ cells, (**B** to **D**) determine the expression of phenotypic markers on CD64^+^F4/80^+^ macrophages. MHCII, major histocompatibility complex II; iNOS, inducible nitric oxide synthase; ARG1, arginase 1; TIM4, T-cell immunoglobulin and mucin domain containing 4; MFI; mean fluorescence intensity. (B) Partial least squares discriminant analysis (PLS-DA) depicting the expression of phenotypic markers on lamina propria macrophages. The left panel reports the score plot, and the right panel reports the variable importance in projection (VIP) scores for the markers investigated and their relative concentrations in the two groups. (C) Expression of CX_3_CR1 on CD64^+^F4/80^+^ macrophages. The top panel is a representative histogram plot. (D) Expression of PD-1 on CD64^+^F4/80^+^ macrophages. The top panel is a representative histogram plot. Results are means ± SEM. *n* = 8 to10 mice per group from two distinct experiments. Statistical differences in (A), (C), and (D) were evaluated using Mann-Whitney test. (**E**) Human monocytes were incubated with 1 nM aspirin and differentiated to macrophages (see Materials and Methods for details). Lipid mediators were identified and quantified using lipid mediator profiling, and differences were evaluated using PLS-DA. Results are representative of four healthy volunteers. The top panel reports the score plot. The bottom panel reports the mediators with the highest VIP scores.

To further explore the mechanisms regulated by aspirin in the observed protection from I-CRC, we then investigated whether aspirin regulated tissue immune cell responses. Given that macrophages play a central role in the onset and progression of I-CRC (*16*, *17*), we first evaluated whether aspirin regulated lamina propria macrophage responses. Aspirin administration reduced the number of macrophages in the lamina propria of mice during I-CRC (Fig. 2Aand fig. S2A). Flow cytometric assessment of phenotypic marker expression on lamina propria macrophages demonstrated a marked change in the phenotype of cells from aspirin-treated mice. This was illustrated by the separation of cell clusters representing cells from aspirin-treated mice and those from vehicle-treated mice in multivariate analysis of macrophage phenotypic marker expression (Fig. 2B). This shift in macrophage phenotype was linked with an increase in the expression of fractalkine receptor CX_3_CR1 (Fig. 2C), which was recently linked with an improved outcome in I-CRC (*18*). Together, these findings demonstrate that aspirin regulates both monocyte-derived macrophage recruitment and phenotype during I-CRC. Moreover, aspirin administration decreased the expression of the immunosuppressive receptor programmed cell death protein-1 (PD-1), which is linked with a negative outcome in I-CRC (Fig. 2D) (*19*, *20*).

Given that macrophages express all the necessary enzymes for SPM biosynthesis, we next assessed whether aspirin regulated lipid mediator production in human monocyte-derived macrophages. Multivariate analysis of lipid mediator profiles from these cells incubated with or without this drug demonstrated that aspirin indeed regulated human macrophage lipid mediator levels and up-regulated the expression of several AT-SPMs including AT-LXB_4_ and AT-RvD1 (Fig. 2E and table S1). These findings demonstrate that aspirin directly regulates both macrophage phenotype and function, reprogramming these cells toward a protective phenotype.

Having observed a change in lipid mediator production by aspirin in human macrophages, including an increase in AT-SPM, we subsequently assessed whether aspirin also regulated the expression of PD-1 in these cells. For this purpose, we prepared human monocyte-derived macrophages and incubated them with conditioned medium obtained from a human CRC cell line, namely, the HT-29 cell line, resulting in increased macrophage PD-1 expression (fig. S3A). Aspirin addition to these cells dose-dependently decreased PD-1 expression (fig. S3A). Recent studies demonstrate that up-regulation of PD-1 in macrophages is linked with a dysregulation in macrophage function, including their ability to uptake apoptotic cells, resulting in increased disease severity (*19*, *20*). Therefore, we next tested whether the down-regulation in PD-1 expression by aspirin was linked with an improvement of macrophage function. Assessment of the kinetics of apoptotic cell uptake by these cells demonstrated that aspirin increased both the rate at which macrophages uptake apoptotic cells and the overall amount of apoptotic cells efferocytosed, as demonstrated by an increase in the fluorescent signal recorded at the 160-min interval (fig. S3, B and C). Notably, these results were replicated using supernatants from a different human CRC cell line, namely, the CACO-2 cell line, where we observed decreased PD-1 expression and increased efferocytosis in macrophages treated with aspirin (fig. S3, D to F).

### Aspirin regulates T cell phenotype and function in I-CRC

**Fig. 3.**
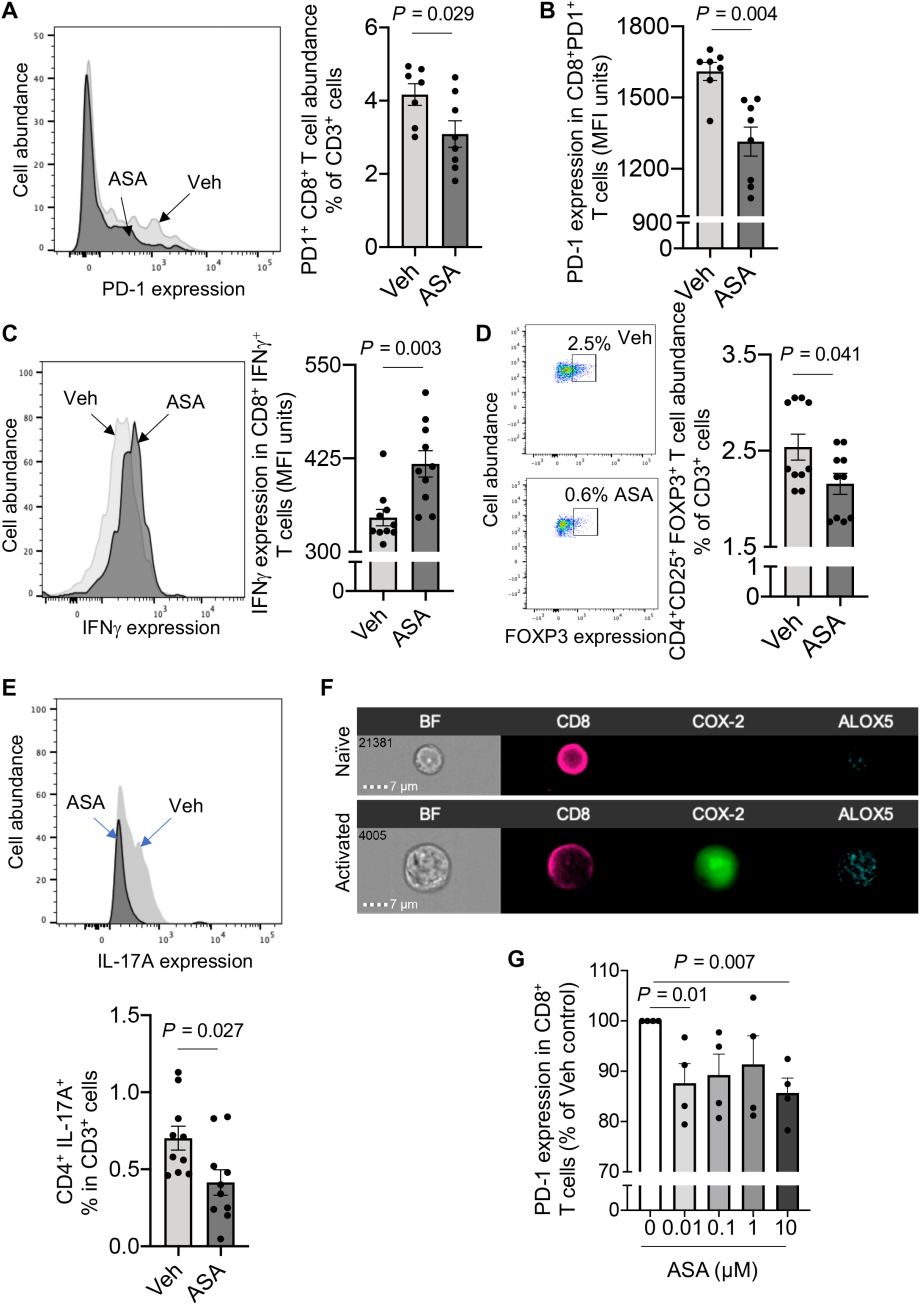
Aspirin restores intestinal T cell responses, up-regulating their effector phenotype during I-CRC. I-CRC was initiated, and mice were treated as in Fig. 1. Colons were harvested, and (**A**) abundance of PD-1–expressing CD8^+^ cells, (**B**) expression of PD-1 in CD8^+^ PD-1^+^ T cells, (**C**) expression of IFNγ in CD8^+^ IFNγ^+^ cells, (**D**) abundance of CD4^+^ FOXP3^+^ T cells, and (**E**) abundance of CD4^+^ IL17A^+^ cells in the colonic lamina propria were determined using flow cytometry. Results are means ± SEM. *n* = 7 to 10 mice per group from two distinct experiments. Statistical differences were evaluated using Mann-Whitney test. (**F**) Human CD8^+^ T cells were incubated with or without anti-CD3/CD28 for 3 days, and the expression of COX-2 and ALOX5 was determined using Imagestream analysis. Results are representative from four healthy volunteers. BF, bright field. (**G**) Human CD8^+^ T cells were incubated with or without CD3/CD28 for 3 days (37°C, 5% CO_2_) and then with the indicated concentrations of aspirin. After 3 days, PD-1 expression was determined using flow cytometry. Results are means ± SEM. *n* = 4 healthy volunteers from two distinct experiments. Statistical differences were evaluated using one-sample *t* test.

Given the role that different T cell populations play in regulating cancer progression, we next investigated whether aspirin also modulated the responses of these cells in mice during I-CRC. We first evaluated whether aspirin regulated the expression of PD-1 on CD8^+^ T cells, because these are central in the identification and killing of tumor cells, and the up-regulation of this receptor is linked with suppression of their cytotoxic functions (*20*). Flow cytometric assessment of lamina propria CD8^+^ T cells demonstrated that aspirin administration significantly reduced both the frequency of cells that expressed PD-1 and the expression of PD-1 per cell (Fig. 3, A and B, and fig. S2B). This decrease in PD-1 expression was linked with a significant up-regulation of the effector cytokine interferon-γ (IFNγ) in these cells (Fig. 3C).

The protective actions of aspirin were not limited to CD8^+^ T cells. We found a significant down-regulation in the expression of the proinflammatory cytokine interleukin-17 (IL-17) in CD4^+^ T cells and a significant reduction in the number of regulatory T cells (T_regs_), which exert immune-suppressive functions, in the lamina propria of mice treated with aspirin (Fig. 3, D and E, and fig. S2B). Notably, these actions were not restricted to murine cells. Incubation of human CD8^+^ T cells, which upon activation rapidly up-regulate the expression of both 5-lipoxygenase (ALOX5) and COX-2 (Fig. 3F), with aspirin led to a dose-dependent decrease in PD-1 expression in these cells (Fig. 3G). Together, these findings demonstrate that aspirin regulates key aspects in CD4^+^ and CD8^+^ T cell responses, which are linked with improved outcomes in I-CRC.

### Aspirin administration leads to a temporal up-regulation of AT-SPM in the colonic mucosa

**Fig. 4.**
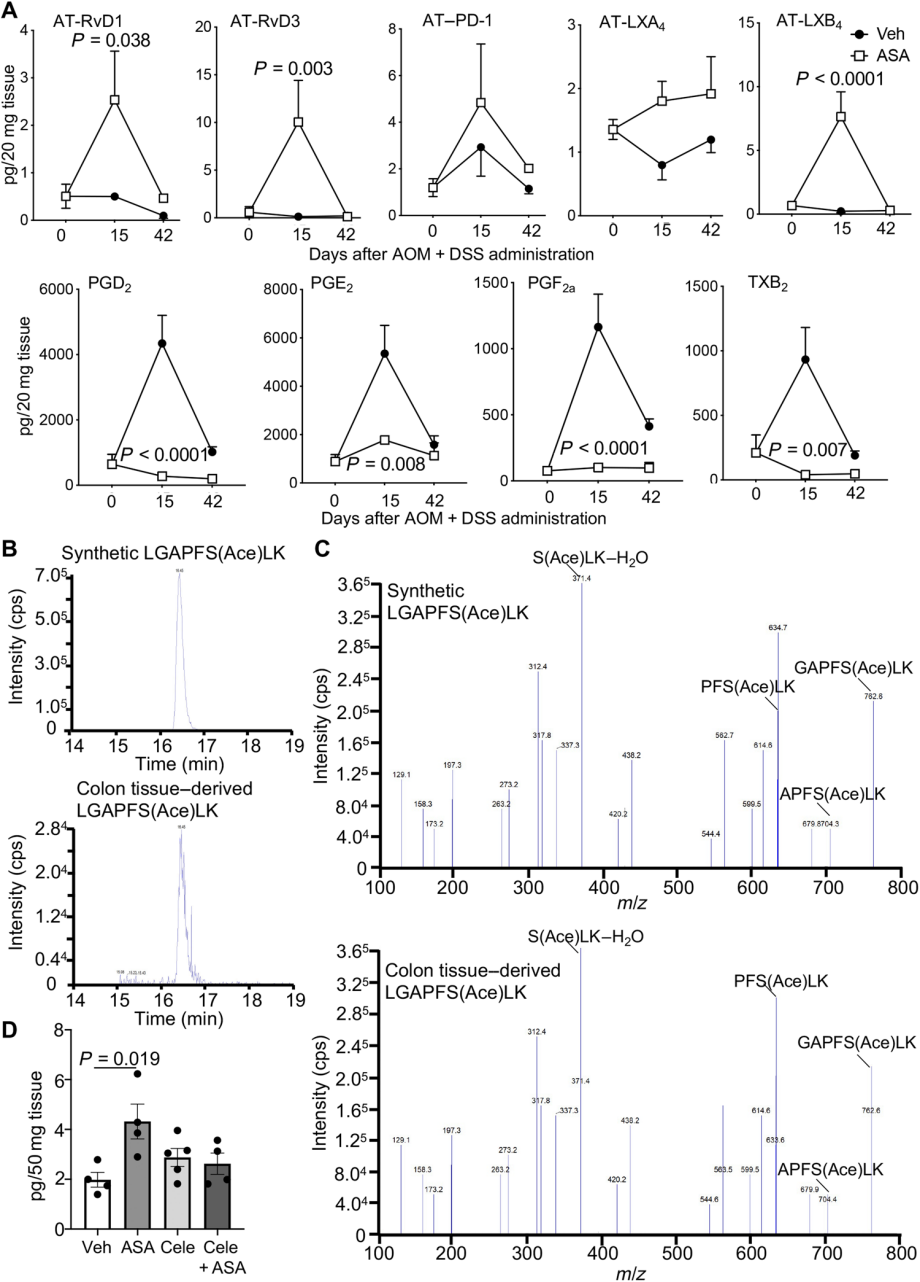
Inhibition of COX-2 activity reverses the ability of aspirin to up-regulate AT-SPM during I-CRC. (**A**) I-CRC was initiated, and mice were treated as in Fig. 1. Colons were harvested at the indicated intervals, and the concentrations of AT-SPM, PGs, and TXB_2_ were determined using LC-MS/MS–based lipid mediator profiling. Results are means ± SEM. *n* = 4 mice per group per interval. Statistical differences were evaluated using two-way analysis of variance (ANOVA) and Sidak’s multiple comparisons post hoc test. (**B** and **C**) I-CRC was initiated as in Fig. 1, and after 7 days, mice were treated with aspirin (43 mg/kg) daily for 7 days. Colon tissues were collected, proteins were separated using gel electrophoresis, the band corresponding to the COX-2 molecular weight was excised, and proteins were digested. Using an LC-MS/MS, we evaluated the presence of an eight–amino acid peptide sequence that encompasses acetylated serine-516. (B) Multiple reaction chromatograms for the ion pair *m*/*z* 438 > 634 for (top) the synthetic peptide used as a reference standard and (bottom) peptides obtained from colonic tissues. (C) MS/MS spectra for the products under the peak eluting at 16.3 min corresponding to (top) the synthetic peptide used as a reference standard and (bottom) peptides obtained from colonic tissues. Results for colonic tissues are representative of three distinct mice. (**D**) I-CRC was initiated as in Fig. 1, and after 1 week, mice were assigned to one of the following groups: vehicle, aspirin (43 mg/kg per day), celecoxib (Cele, 30 mg/kg per day), or aspirin + celecoxib. After 1 week, colons were collected, and AT-SPM concentrations were determined using LC-MS/MS. Results are from *n* = 4 to 5 mice per group. Statistical differences were evaluated using Kruskal-Wallis test and Dunn’s multiple comparisons post hoc test.

To establish the mechanism by which aspirin exerts its immune-protective actions, we assessed the temporal biosynthesis of AT-SPM in mice, given that these mediators are known to exert potent immunoregulatory actions (*9*, *21*). Here, we found that aspirin administration rapidly increased the biosynthesis of AT-RvD1, AT-RvD3, AT–PD1, AT-LXA_4_, and AT-LXB_4_ in colonic tissues, which reached a maximum 1 week after treatment initiation (Fig. 4A). This increase in AT-SPM was coupled with a significant reduction in the immunosuppressive mediators PGE_2_, PGD_2_, and the prothrombogenic TXA_2_, measured as its stable further metabolite TXB_2_ (Fig. 4A). Notably, this shift in mediator production in mice treated with aspirin was not linked with a down-regulation of COX-2 expression, 1 week after treatment initiation, in both colonic tissues and lamina propria macrophages from mice treated with aspirin when compared with those receiving vehicle alone (fig. S4, A and B). This suggests a shift in the catalytic activity of COX-2 in aspirin-treated mice, lending further support to the role of AT-SPM in mediating the protective actions of aspirin in I-CRC.

To further dissect the biosynthetic pathway leading to the observed increase in AT-SPM, we next evaluated whether aspirin administration led to the acetylation of COX-2 in mouse colonic tissues. For this purpose, intestinal inflammation was initiated using AOM and DSS. After 1 week of DSS, mice were treated daily with aspirin for 7 days, and colon tissues were collected. COX-2 acetylation at serine-516 was then evaluated using LC-MS/MS whereby the protein was digested to yield a peptide with the following amino acid sequence -LGAPFSLK- that contains serine-516 (*22*). Results from these studies demonstrate the presence of acetylated COX-2 in these tissues, as illustrated by the presence of a peak within the multiple reaction monitoring transition of mass/charge ratio (*m*/*z*) 438 > 634, which eluted at the same retention time as that of the corresponding acetylated synthetic peptide (Fig. 4B). Furthermore, assessment of the MS/MS spectrum for the ions under this peak supported the presence of the acetyl group on serine-516 as demonstrated by the presence of an ion at an *m*/*z* of 634 that corresponds to a fragment of the peptide containing the acetylated serine, i.e., PFS(Ace)LK (Fig. 4C).

Having established the presence of acetylated COX-2 in colonic tissues after aspirin administration, we next queried whether this enzyme was responsible for the observed increases in AT-SPM concentrations observed in mice given aspirin. For this purpose, we assessed whether inhibition of COX-2 activity using the COX-2–selective inhibitor celecoxib would block the observed increase in AT-SPM concentrations. Here, inflammation was initiated as detailed above, and then, mice were treated with vehicle or aspirin with or without celecoxib. Intriguingly, while aspirin administration was observed to increase overall AT-SPM concentrations, administration of celecoxib decreased this aspirin-mediated up-regulation of AT-SPM (Fig. 4D). Together, these findings lend support to the hypothesis that aspirin acetylates COX-2 in mouse colonic tissues promoting the up-regulation of AT-SPM.

### Inhibition of AT-SPM biosynthesis reverses the protective actions of aspirin in I-CRC

**Fig. 5.**
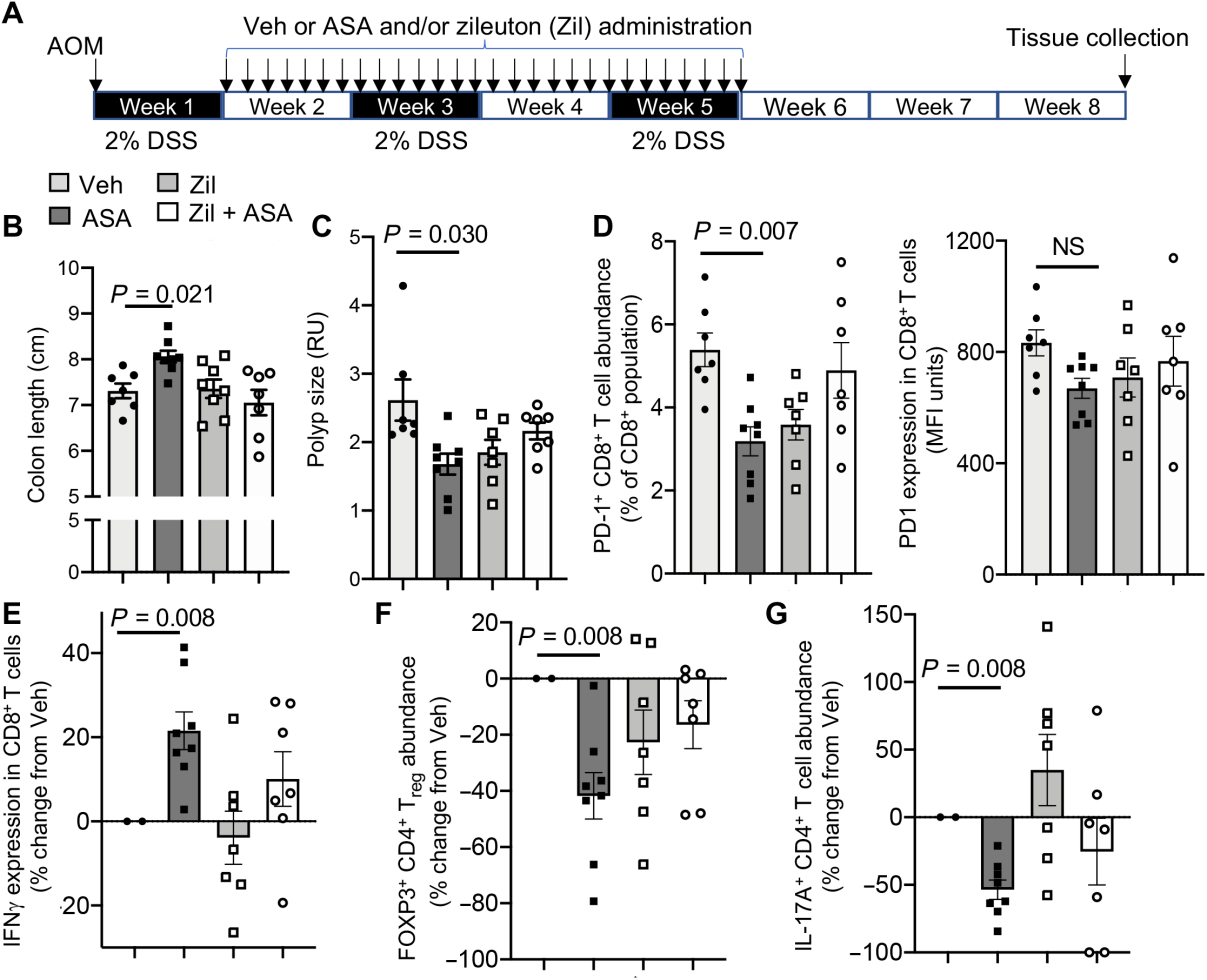
Inhibition of AT-SPM biosynthesis reverses the protective actions of aspirin in I-CRC. (**A** to **G**) I-CRC was initiated by administrating AOM (7.5 mg/kg via intraperitoneal injection) and DSS (2% in the drinking water). After 7 days, mice were treated with aspirin (43 mg/kg), zileuton (Zil; 10 mg/kg), aspirin together with zileuton, or vehicle via oral gavage, daily for a 4-week period. (A) Experimental timeline, (B) colon length, and (C) polyp area were determined at the end of the experiment. RU, Relative Units. (D to G) Lamina propria leukocytes were liberated, and (D) PD-1 together with (E) IFNγ expression in CD8^+^ cells were determined using flow cytometry. (F) Abundance of FOXP3^+^ CD4^+^ T cells and (G) abundance of IL17A^+^ CD4^+^ T cells was determined using flow cytometry. Results are means ± SEM. *n* = 8 mice per group from two distinct experiments. Statistical differences were evaluated using Wilcoxon signed-rank test or one-way ANOVA and Dunn’s multiple comparisons post hoc test. NS, not statistically significant.

We next sought to evaluate the role of AT-SPM in mediating the observed protective actions of aspirin. Because AT-RvD1, AT-RvD3, AT-LXA_4_, and AT-LXB_4_ are produced via the interaction of COX-2 and ALOX5 (*7*), we used a pharmacological approach to inhibit AT-SPM production by inhibiting ALOX5 activity (Fig. 5A). This strategy was used to obviate for any protective activities that might be derived from the inhibition of COX-2–derived prostanoid production (*12*). We then assessed whether aspirin retained its protective actions. Administration of the ALOX5 inhibitor zileuton decreased the expression of AT-RvD1, AT-RvD3, AT-LXA_4_, and AT-LXB_4_ without significantly changing prostanoid levels when compared with mice treated with aspirin alone (fig. S4C). These changes were linked with a reversal of the protective actions of aspirin as demonstrated by an increase in colon shortening and polyp area when compared with mice given aspirin alone (Fig. 5, B and C). Flow cytometric assessment of PD-1 expression in macrophages and CD8^+^ T cells demonstrated that administration of zileuton blunted the ability of aspirin to down-regulate the expression of this immunosuppressive receptor (Fig. 5D). Zileuton also limited the ability of aspirin to increase IFNγ in these cells (Fig. 5E). These observations were linked with a blunting in the ability of aspirin to regulate IL-17A expression in CD4^+^ T cells and T_reg_ abundance in the colonic mucosa of mice that were also treated with zileuton, when compared with mice administered aspirin alone (Fig. 5, F and G). Together, these findings support the role of AT-SPM in mediating the immune-protective actions of aspirin in I-CRC.

### Loss of the murine homolog of *Alx/Fpr2* blunts the protective actions of aspirin in I-CRC

To further explore the role of endogenous AT-SPM in mediating the immune-protective actions of aspirin, we next assessed whether aspirin retained its ability to reduce I-CRC and regulate immune response in mice lacking the *Alx/Fpr2* receptor, given that this is the cognate receptor for AT-RvD1, AT-RvD3, and AT-LXA_4_ (*23*). Here, we found that, despite aspirin treatment, there were no differences in disease severity, polyp area, macrophage phenotype, and the expression of PD-1 by lamina propria macrophages and CD8^+^ T cells (fig. S5, A to F). Notably, we observed that, in these mice, aspirin administration failed to regulate the expression of CX_3_CR1 in lamina propria macrophages, that of IFNγ in CD8^+^ T cells, IL-17A expression in CD4^+^ T cells, and T_reg_ abundance (fig. S5, E to I). Together, these findings demonstrate a role for *Alx/Fpr2* in mediating the immune-protective actions of aspirin in I-CRC.

### AT-SPM are immunoregulatory in I-CRC

**Fig. 6.**
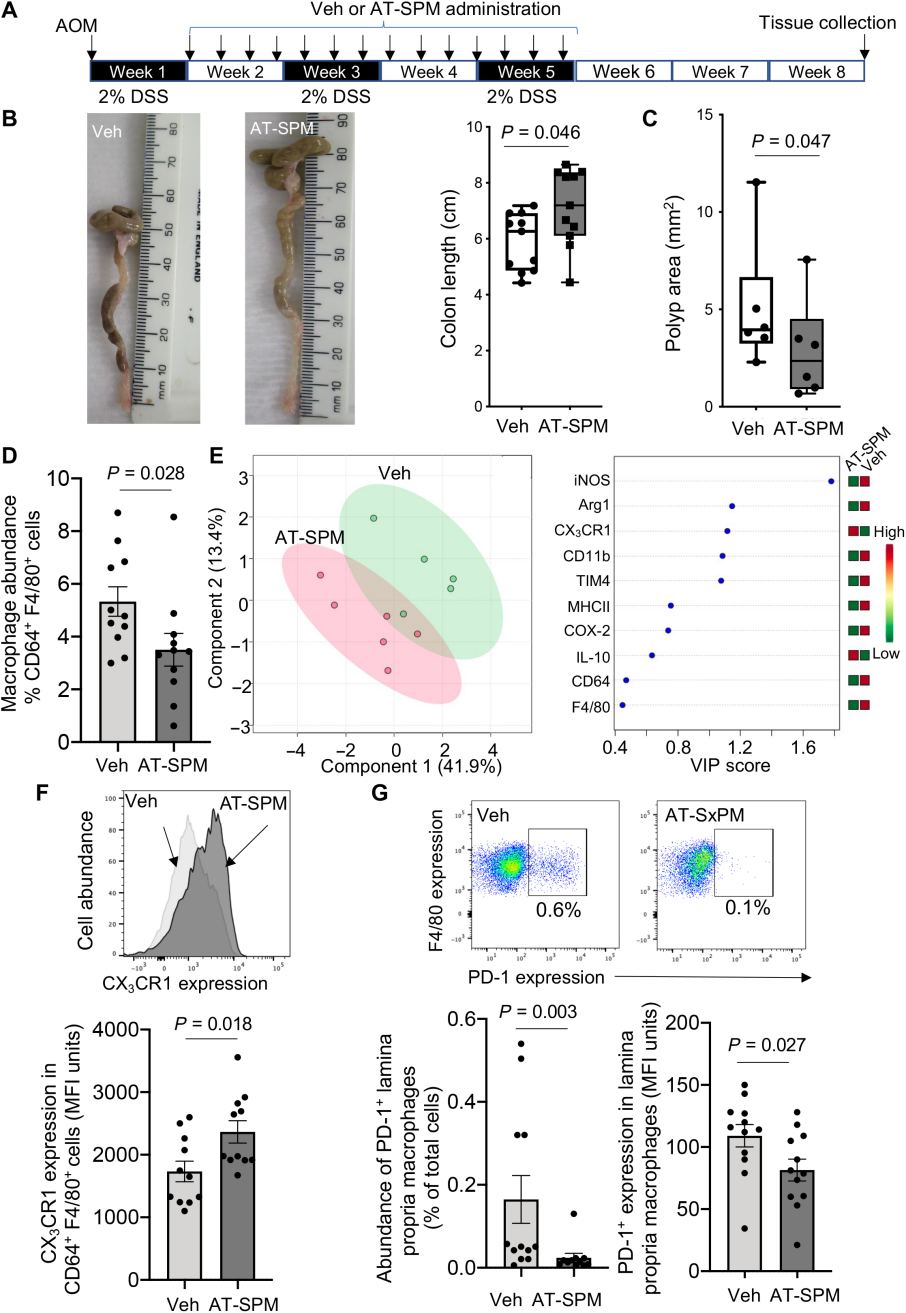
AT-SPMs protect against I-CRC decreasing PD-1 and up-regulating CX_3_CR1 expression in lamina propria monocyte-derived macrophages. I-CRC was initiated by administrating AOM (7.5 mg/kg via intraperitoneal injection) and DSS (2% in the drinking water). After 7 days, mice were treated on alternative days with a combination of AT-RvD1, AT-RvD3, AT–PD1, AT-LXA_4_, and AT-LXB_4_ (15 ng per mouse each; AT-SPM), or vehicle via intraperitoneal injection, on alternative days for a 4-week period. (**A**) Experimental timeline, (**B**) representative images (left), colon length (right), and (**C**) polyp area were determined. Results are means ± SEM. *n* = 11 for (B) and 6 for (C). (**D** to **G**) Lamina propria leukocytes were liberated, and (D) the abundance of CD64^+^F4/80^+^ cells was determined using flow cytometry. (E) Expression of lineage markers was determined using flow cytometry on monocyte-derived macrophages, and results were interrogated using PLS-DA. Left: Score plot. Right: VIP scores for each of the lineage markers investigated. (F) Expression of CX_3_CR1 and (G) PD-1 on monocyte-derived macrophages was determined using flow cytometry (top insets are representative flow cytometry plots). Results are means ± SEM. *n* = 12 mice per group from two independent experiments. Statistical differences were evaluated using Mann-Whitney test. Photo credit: Roberta De Matteis and Shani Austin-Williams, QMUL.

To further underpin the role of AT-SPM in the observed immune-protective actions exerted by aspirin in I-CRC, we next investigated whether administration of the AT-SPM that were up-regulated by aspirin in colonic tissues during I-CRC would recapitulate the protective actions observed with aspirin treatment. Administration of a mixture of these mediators at a dose as low as 15 ng each per mouse on alternate days was found to protect against colon shortening and reduce polyp area to a similar extent as aspirin (Fig. 6, A to C).

**Fig. 7.**
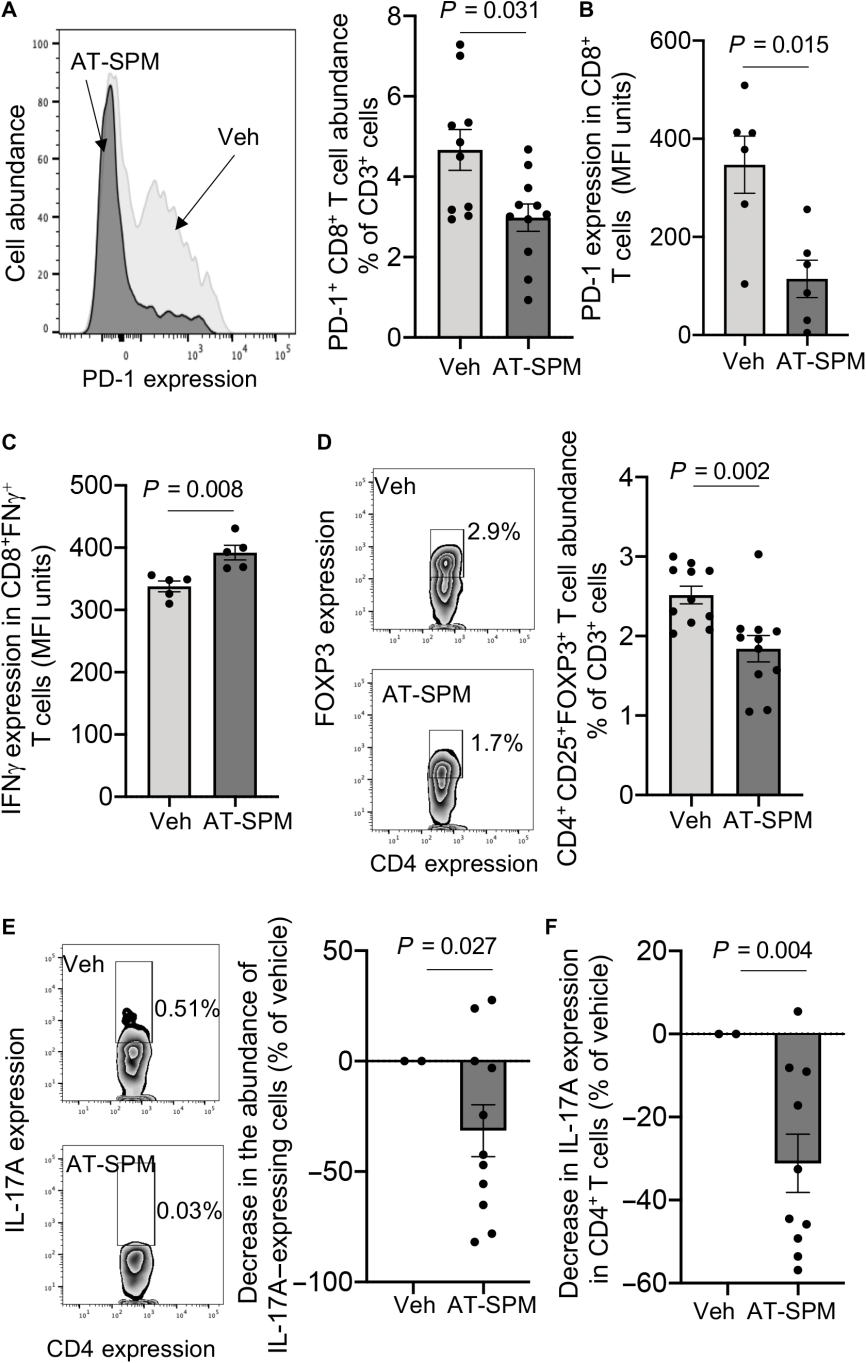
AT-SPMs restore intestinal CD8^+^ T cell responses during I-CRC. I-CRC was initiated, and mice were treated as in Fig. 5. Colons were harvested, and (**A**) the abundance of PD-1–expressing CD8^+^ cells (left panels display representative flow cytometry plots), (**B**) the expression of PD-1 in CD8^+^ PD-1^+^ T cells, (**C**) the expression of IFNγ in CD8^+^ INFγ^+^ cells, (**D**) the abundance of CD4^+^ FOXP3^+^ T cells (left panels display representative flow cytometry plots), (**E**) the abundance of CD4^+^ IL-17A^+^ cells (left panels display representative flow cytometry plots), and (**F**) the expression of IL-17A in CD4^+^ T cells in the colonic lamina propria were determined using flow cytometry. Results are means ± SEM. *n* = 6 to 10 mice per group from two distinct experiments. Statistical differences were evaluated using Mann-Whitney test.

Flow cytometric analysis demonstrated that these molecules also regulated both the trafficking and phenotype of macrophages collected from colonic tissues of mice given AT-SPM, when compared with those collected from colons of mice treated with vehicle alone (Fig. 6, D and E). This shift in macrophage phenotype was linked with an up-regulation of CX_3_CR1 (Fig. 6F). We also found that AT-SPM reduced the expression of PD-1 on both macrophages and CD8^+^ T cells and up-regulated the expression of IFNγ on lamina propria CD8^+^ T cells (Figs. 6G and 7, A to C). The protective actions of AT-SPM were also observed on CD4^+^ T cells, where we observed a reduction in immunosuppressive T_regs_ and a decrease in the expression of IL-17A (Fig. 7, D to F). Together, these findings demonstrate that AT-SPMs recapitulate the immune-directed actions of aspirin in I-CRC.

To determine whether the regulation of macrophage responses observed in vivo was via direct regulation of cellular responses, we next evaluated the ability of AT-SPM to regulate PD-1 expression in human monocyte-derived macrophages incubated with conditioned medium from human CRC cells. Flow cytometric assessment demonstrated a dose-dependent down-regulation in the expression of this receptor on macrophages, with AT-LXA_4_ giving the largest reduction in PD-1 expression (fig. S6A). This down-regulation was linked with an increase in the ability of macrophages to efferocytose apoptotic cells. In these experiments, AT-LXA_4_ and AT-RvD1 displayed the highest potency at up-regulating macrophage efferocytosis as demonstrated by an ~60% increase in macrophage-associated fluorescent signal at the 160-min interval (fig. S6, B and C). Similar findings were made when cells were incubated with supernatants from CACO-2 cells, where AT-SPM reduced macrophage PD-1 expression and up-regulated their ability to uptake apoptotic cells when compared with cells incubated with vehicle alone (fig. S7). Last, we assessed whether AT-SPM also exerted direct actions on CD8^+^ T cells. Flow cytometric analysis of CD8^+^ T cells demonstrated that AT-LXA_4_, AT-LXB_4_, and AT–PD1 dose-dependently reduced PD-1 expression in these cells (fig. S8A). Together, these findings demonstrate that AT-SPMs regulate key immune-protective responses that are linked with improved outcomes in I-CRC.

## DISCUSSION

The present study establishes a role for AT-SPM in mediating the immune regulatory actions of aspirin in I-CRC. Aspirin administration up-regulated AT-SPM concentrations, reduced polyps, down-regulated the expression of tumorigenic genes, regulated macrophage trafficking in colorectal tissues, and reduced the expression of the immunosuppressive receptor PD-1 in both macrophages and CD8^+^ T cells. Aspirin also regulated tissue metabolism, reducing the levels of several glycolytic intermediates. Inhibition of AT-SPM formation or deletion of the proresolving receptor *Alx/Fpr2* reversed the protective actions of aspirin. Notably, administration of AT-SPM replicated the immunoregulatory actions of aspirin, modulating macrophage phenotype and trafficking, reducing the expression of PD-1 in both macrophages and CD8^+^ T cells, and up-regulating the expression of IFNγ in CD8^+^ T cells and the clearance of apoptotic cells by macrophages (see fig. S8B).

Aspirin is one of the most widely used medications and is listed on the WHO Model List of Essential Medicines. It is appreciated for its analgesic and anti-inflammatory actions as well as for its ability to reduce incidence of myocardial infarction and stroke (*24*, *25*). The main mechanism that is attributed for these biological actions is its ability to inhibit COX activity and therefore the formation of PGs, in particular PGE_2_, which has potent pyretic and vasodilatory actions and TxA_2_, which is a potent agonist of platelet aggregation (*24*, *25*). Recent studies uncovered a previously unappreciated biological role for this drug. Aspirin, by acetylating COX-2, shifts the catalytic activity of this enzyme that, instead of converting arachidonic acid to PGG_2_, converts this substrate to 15*R*-hydroperoxyicosatetraenoic acid or docosahexaenoic acid to 17*R*-hydroperoxydocosahexaenoic acid. Both are precursors in the biosynthesis of AT-SPM (*7*, *8*). These molecules retain the potent biological actions of the lipoxygenase-derived SPM and display enhanced metabolic stability given that the *R* chirality of the hydroxyl group makes them less susceptible to further conversion by 15-PG dehydrogenase (*26*). In the present studies, we found that in mouse colonic tissues, aspirin up-regulated the concentrations of AT-SPM and down-regulated tissue prostanoids in a time-dependent manner.

Studies investigating the ability of aspirin to regulate AT-SPM production in humans demonstrated that low-dose aspirin displays the greatest ability to up-regulate the concentrations of AT-LXA_4_ (or 15-epi-LXA_4_) in peripheral blood of healthy volunteers. At the intermediate dose, aspirin was still able to up-regulate plasma concentrations of this mediator, whereas no increase was observed at the highest dose administered when compared to placebo. Notably, assessment of plasma TXB_2_ concentrations demonstrated a significant decrease in plasma concentrations of this molecule at all three doses tested when compared with placebo (*27*). These results suggest that, while aspirin’s ability to acetylate COX-1 in platelets does not change with the doses tested, this drug is more effective at switching the catalytic activity of COX-2 at the lower doses. In the present study, we found that aspirin displayed its greatest protective actions on colonic inflammation at the intermediate equivalent dose. It should be noted that the dose conversion between humans and mice was based on body surface area and does not reflect differences in biochemical, metabolic, and pharmacokinetic properties between the two species. Published findings support the observation that the low dose tested may not fully replicate the pharmacological properties of aspirin as observed in humans, including a blunted ability to block TXB_2_ production in mouse blood (*4*). Thus, these observations may explain the apparent difference between our present findings and those reported in humans.

During I-CRC, a shift occurs in the colonic tissue metabolic profile, leading to an increase in tissue glycolysis. This is linked with a reduced availability of glucose and a switch in the metabolic activity of immune cells that is thought to be, in part, responsible for the up-regulation of immune-suppressive pathways such as the PD-1/Programmed death-ligand 1 (PDL1) pathway in immune cells (*14*, *15*, *28*). Recent studies demonstrate that manipulation of metabolic pathways may reduce cancer progression by ameliorating immune responses (*14*, *15*, *28*). In the present investigations, we found that administration of aspirin reduced the concentrations of select glycolytic intermediates in mouse colonic tissues. This was linked with a down-regulation of hexokinase-2, a key enzyme in the glycolytic pathway that converts glucose to glucose-6-phosphate resulting in the retention of this metabolite inside the cell. Notably, increased expression of this enzyme in colorectal carcinoma tissue is linked with a poor prognosis (*29*).

In inflammatory bowel disease (IBD), macrophages are thought to acquire a proinflammatory phenotype and actively participate in the perpetuation of tissue inflammation (*30*). As IBD progresses to CRC, the tumors produce factors that alter the macrophage phenotype, up-regulating the expression of immunosuppressive molecules. In this context, up-regulation of PD-1 in gut macrophages impairs their ability to clear apoptotic cells, a mechanism linked with cancer progression (*19*). Underscoring the importance of this mechanism in this pathogenic process are findings from recent studies that demonstrate that inhibition of PD-1 in myeloid cells, including macrophages, represents a key mechanism of antitumor immunity mediated by PD-1 blockade in experimental systems (*31*). Notably, specific macrophage subsets are also associated with improved prognosis in CRC. Elevated infiltration of macrophages expressing CD80, CD86 (*32*), and CX_3_CR1 (*18*) are all associated with improved outcomes. The present findings demonstrate that aspirin reprograms macrophage phenotype, up-regulating the expression of CX_3_CR1 and down-regulating PD-1. These biological actions were abolished when either AT-SPM production was inhibited or the AT-SPM receptor *Alx/Fpr2* was knocked out in mice. Furthermore, administration of AT-SPM replicated the macrophage-directed regulatory actions of aspirin. Notably, the observed changes in macrophage phenotype and PD-1 expression exerted by AT-SPM and aspirin were functionally relevant given that they were also linked with an improved ability of cells to uptake apoptotic cells. These findings are in accord with published findings demonstrating that aspirin and SPM regulate macrophage responses, including their ability to clear apoptotic cells and cellular debris (*21*).

T cells are also implicated in CRC pathogenesis. CD8^+^ T cells are central in antitumor responses; however, their responses become impaired during tumor progression. This phenomenon is linked with the up-regulation of immunosuppressive molecules on these cells, including PD-1. This leads to a reduction in their ability to kill tumorigenic cells, facilitating tumor progression and carcinogenesis (*20*). Results from the present studies support a role for AT-SPM in observed regulation of PD-1 expression in CD8^+^ T cells given that inhibition of AT-SPM formation (using zileuton) or activity (knockout of *Alx/Fpr2*) reversed its ability to down-regulate the expression of this receptor and increased IFNγ expression in these cells. Notably, the protective actions of aspirin and AT-SPM extend beyond CD8^+^ T cells. Aspirin also decreased the expression of IL-17A in CD4^+^ T cells and the abundance of T_regs_ in colonic tissues during I-CRC. These T cell subsets are, respectively, linked with supporting angiogenesis and tissue glycolysis as well as immune suppression.

While the integrated approach used herein supports the potential role of AT-SPM in mediating the protective activities of aspirin in I-CRC, there are some limitations that should be considered when evaluating these findings and their translation potential. Results from published studies demonstrate that aspirin up-regulates plasma AT-SPM in human peripheral blood (*27*). In addition, findings from the present studies demonstrate that (i) aspirin up-regulates AT-SPM formation in human monocyte-derived macrophages, (ii) administration of aspirin is linked with COX-2 acetylation in mouse colonic tissues during I-CRC, and (iii) inhibition of this enzyme limits AT-SPM formation. Nonetheless, human studies will need to establish whether aspirin also acetylates COX-2 in human colons, including human colonic epithelial cells and lamina propria macrophages, to up-regulate AT-SPM formation. They will also need to detail where within the disease course the present findings are most relevant, especially in light of clinical findings demonstrating that the greatest benefit derived from aspirin is primarily observed in those patients taking aspirin for more than 5 years (*2*, *33*, *34*). Future studies will also need to evaluate whether the proposed mechanism is active in limiting immune responses in select disease settings or widely applicable to inflammation-driven tumorigenesis where aspirin has been observed to play protective roles.

Together, the present findings demonstrate a central role for proresolving pathways in mediating the immunomodulatory actions of aspirin during I-CRC. These findings are in accord with published literature demonstrating that aspirin may be more effective in preventing sporadic CRCs that overexpress COX-2 (*35*). They also offer a novel immune-directed mechanism that is complementary to existing proposed anti-inflammatory activities of this drug, including the inhibition of PGE_2_ and TXA_2_ formation, which contribute to limiting inflammation-mediated tumorigenesis. They also uncover novel mechanisms elicited by AT-SPM in regulating both innate and adaptive immune responses during I-CRC that may be applicable to other cancers where PD-1 plays a central role in disease onset and/or progression, thereby the present findings supporting the utility of SPM-based therapeutics in both cancer prevention and treatment. These treatments may be especially useful in settings where aspirin use is suggested to increase the risk of bleeding and ulceration, thus limiting it use, for example, in patients with IBD.

## MATERIALS AND METHODS

### Inflammation-associated colon cancer

Male 11-week-old specific pathogen–free C57BL/6 mice were procured from Charles River Laboratories UK (Kent, UK). Mice were maintained in individually ventilated cages on a standard chow pellet diet and had access to water ad libitum, with a 12-hour light/12-hour dark cycle. All animal experiments were performed strictly in accordance with the UK Home Office regulations (Guidance on the Operation of Animals, Scientific Procedures Act) and Laboratory Animal Science Association Guidelines (Guiding Principles on Good Practice for Animal Welfare and Ethical Review Bodies) and according to protocols detailed in a UK Home Office–approved protocol (P998AB295).

I-CRC was initiated as detailed previously (*11*). Briefly, mice were administered AOM (7.5 mg/kg) via intraperitoneal injection on day 0. Mice were then given 2% DSS in drinking water for 1 week, followed by 1 week of normal drinking water. This was followed by two more cycles of DSS/normal water and by an additional 3 weeks of normal water. At the end of the experiments, disease progression was assessed as detailed below. In select experiments, mice were given aspirin at 10, 43, and 86 mg/kg (equivalent to 75, 325, and 650 mg in humans using body surface normalization) with or without zileuton (10 mg/kg; dissolved in water) or water (alone) daily via oral gavage starting at the end of the first week of DSS dosing for 4 weeks. After 8 weeks, intestines were collected, and disease progression was assessed. Disease severity was determined by assessing shortening in colon length. This was measured using the “freehand line” tool on ImageJ and drawing a line down the center portion of the tissue. Colon length was then obtained using the measure tool in ImageJ. This value was then normalized using the scale included in each of the images taking into account differences related to the distance between the camera objective and the tissue between images. Polyp area was determined by measuring the overall area occupied by polyps in the distal colon region using ImageJ software. Area was then normalized as detailed above for colon length.

In designated experiments, mice deficient in *Alx/Fpr2* were administered AOM at 7.5 mg/kg via intraperitoneal injection on day 0. Given that *Alx/Fpr2* mice displayed increased susceptibility to colitis, as also reported by others (*35*), with 100% of the mice reaching severity limits before the end of the experiment, the DSS dose in these experiments was reduced to 1%. This afforded disease severity that was comparable to that observed in wild-type mice. We followed the same DSS/normal water treatment regime as described above and detailed in fig. S5. Mice were also administered aspirin (43 mg/kg) or water daily via oral gavage starting at the end of the first week of DSS dosing and terminating after the last DSS cycle on week 6. After 8 weeks from the start of AOM/DSS, intestines were collected, and disease progression was assessed. Disease severity was determined as above.

In select experiments, I-CRC was initiated as detailed above, and after 1 week, mice were randomized to one of four treatment groups and treated with either celecoxib (30 mg/kg per day) or vehicle [phosphate-buffered saline (PBS)], followed by aspirin (43 mg/kg per day) or vehicle daily for 7 days. At the end of the experiment, colon tissues were harvested, and AT-SPM expression together with the presence of acetylated COX-2 were evaluated as detailed below using LC-S/MS.

### Isolation of lamina propria leukocytes

For isolation of lamina propria lymphocytes, colons were excised, washed in 2% fetal bovine serum/Hanks’ balanced salt solution (HBSS), and then cut into small segments. Segments were shaken in 2 mM EDTA/HBSS for 15 min at 37°C and then washed with HBSS, followed by further shaking in 2 mM EDTA/HBSS for 15 min at 37°C and washing with HBSS. Next, tissues were digested in collagenase type VIII (1 mg/ml) (Sigma-Aldrich, UK) and deoxyribonuclease I (10 μg/ml) (Sigma-Aldrich, UK) in complete RPMI 1640 by shaking at 37°C for 45 min and passed through 70-μm strainers, and single cells were centrifuged. Tissue digest was then passed again through 70-μm strainers.

### Human primary leukocytes

Studies involving human primary cells were approved by the Queen Mary Research Ethics Committee, London, UK (QMREC 2014:61). To obtain human monocyte-derived macrophages, leukocyte cones were procured from the National Health Service Blood and Transplant Bank. Peripheral blood mononuclear cells (PBMCs) were isolated using Histopaque 1077 (MilliporeSigma) density centrifugation, and peripheral blood monocytes were incubated with macrophage colony-stimulating factor (M-CSF) (20 ng/ml; R&D Systems, UK) with or without the indicated doses of aspirin for 7 days. Incubations were then quenched using ice-cold methanol, and lipid mediator concentrations were determined as detailed below.

In select experiments, human monocytes were incubated with M-CSF (20 ng/ml; R&D Systems, UK). After 7 days, the cells were incubated with conditioned medium obtained from HT-29 cells for 8 hours. Cells were then incubated with the indicated concentrations of aspirin or AT-SPM for 16 hours, and the expression of PD-1 was determined using flow cytometry as detailed below. To determine whether aspirin and AT-SPM regulate efferocytosis, cells (3.5 × 10^4^ per well) were seeded in 96-well plates. Macrophages were allowed to adhere overnight and then incubated with conditioned medium from HT-29 cells or CACO-2 cells for 8 hours. Cells were then incubated with the indicated concentrations of aspirin or AT-SPM for 16 hours, and efferocytosis of apoptotic cells was determined. Here, apoptotic fluorescently labeled cells were generated from human promyelocytic HL-60 cells that were seeded at 1 × 10^6^ cells/ml in 35-mm plates, irradiated with ultraviolet C light (254 nm) for 15 min, and incubated at 37°C for 2 hours. Apoptosis induction was verified by flow cytometry using the APC Annexin V Apoptosis Detection Kit with PI (BioLegend), where annexin V–positive and annexin V/PI–double positive cells were considered to be apoptotic. Apoptotic HL-60 cells were washed with PBS and labeled with 1 μM pHrodo Red succinimidyl ester (Invitrogen) in PBS for 30 min at room temperature. Macrophages were stained with CellBrite Green (Biotium) for 1 hour at 37°C to visualize cell membranes and washed with RPMI 1640. Apoptotic pHrodo Red–labeled HL-60 cells were then added to the macrophages in a 5:1 ratio (apoptotic HL-60:macrophage), and the increase in pHrodo Red signal over time in macrophages (representing apoptotic cell efferocytosis by macrophages) was quantified using a Zeiss Celldiscoverer 7 high-content imaging system.

### Human CD8 T cell incubations

CD8^+^ T cells were isolated from human PBMCs, obtained as detailed above, using the EasySep Human CD8^+^ T Cell Isolation Kit (STEMCELL Technologies) according to the manufacturer’s instructions. Isolated CD8^+^ cells were cultured in X-VIVO 15 media (Lonza) at a concentration of 10^5^ cells/ml with an equal number of Dynabeads Human T-Activator CD3/CD28 for T Cell Expansion and Activation (Gibco) and recombinant human IL-2 (20 ng/ml; BioLegend). Cells were cultured for 6 days, with media refreshed at day 3. In designated experiments, cells were incubated with aspirin (10 to 0.01 μM), AT-SPM (0.1 or 1 nM), or vehicle for 3 days at 37°C in 5% CO_2_. At the end of the incubations, cells were harvested, and PD-1 expression was investigated using flow cytometry.

### Real-time polymerase chain reaction

Colon tissue was homogenized using a Precellys 24 tissue homogenizer and Lysing Matrix E tubes (MP Biomedicals) containing RLT buffer (QIAGEN) with β-mercaptoethanol (MilliporeSigma). This was followed by isolation of total RNA using the RNeasy Mini Kit (QIAGEN) as per the manufacturer’s instructions. Next, reverse transcription of total RNA was performed with deoxynucleotide triphosphates, Oligo(dT)20 Primers, RNaseOUT, and SuperScript IV reverse transcriptase (all Invitrogen, Thermo Fisher Scientific) as per the manufacturer’s instructions. Real-time polymerase chain reaction was carried out using PowerUp SYBR Green Master Mix (Thermo Fisher Scientific) on an Applied Biosystems 7900HT Fast Real-Time PCR System (Thermo Fisher Scientific). Next, the expression of the genes of interest was normalized against that of housekeeping gene 18*S* ribosomal RNA (rRNA). The following primers were used: 18*S* rRNA, 5′-CATTCGAACGTCTGCCCTATC-3′ (Fw) and 5′-CCTGTGCCTTCCTTGGA-3′ (Rev); *Tff3*, Mm_Tff3_1_SG QuantiTect Primer Assay; *cMyc*, Mm_Myc_1_SG QuantiTect Primer Assay; *Tpi1*, Mm_Tpi1_1_SG QuantiTect Primer Assay; *Pten*, Mm_Pten_1_SG QuantiTect Primer Assay; and *Hk2*, Mm_Hk2_1_SG QuantiTect Primer Assay (QIAGEN).

### Immunofluorescence staining

Immunofluorescence staining of colon tissue was performed as in (*36*). In short, 5-μm sections were cut from Carnoy’s solution-fixed, paraffin-embedded tissues and then deparaffinized with xylene and rehydrated, and antigens were retrieved by boiling in 10 mM sodium citrate (pH 6.0). Tissues were blocked in 10% normal goat serum and then incubated with fluorescently labeled anti-Ki67 antibody [1:50; Ki67 monoclonal antibody (SolA15), eFluor 660, eBioscience] in Dako antibody diluent (Agilent Technologies) overnight at 4°C and mounted with ProLong Gold Antifade mountant with 4′,6-diamidino-2-phenylindole (Thermo Fisher Scientific). Ki67-expressing cells were visualized and imaged with a Zeiss LSM 710 microscope, and Ki67-positive cells per crypt were quantified.

### Flow cytometry

Lamina propria leukocytes, human monocyte-derived macrophages, and T cells were incubated with Fc-blocking antibodies and then with 0.1% LIVE/DEAD stain for 20 min on ice. Cells were then stained for 30 min on ice with fluorescently labeled antibodies (see table S2 for details) in Dulbecco’s PBS^−/−^ containing 0.02% bovine serum albumin and 1% Fc-blocking immunoglobulin G (IgG) and then fixed with fixation/permeabilization solution (Cytofix/Cytoperm Kit, BD Biosciences) following the manufacturer’s instructions. Intracellular staining was performed after permeabilization using permeabilization buffer (eBioscience) by incubating cell suspension for 30 min at room temperature with fluorescently labeled antibodies in permeabilization buffer. Staining was evaluated using an LSRFortessa (BD Biosciences, UK) cell analyzer and FlowJo software (FlowJo LLC, USA) for analysis.

### Image stream

Human CD8^+^ cells were stained using fluorescently labeled antibodies as described above. Staining was then evaluated using an ImageStream X MK2, using IDEAS (Image Data Exploration and Analysis Software, version 6.0).

### Targeted lipid mediator profiling

Colons were harvested from mice and immediately placed in 1 ml of ice-cold methanol containing deuterium-labeled internal standards (500 pg for d_4_-PGE_2_, d_8_–5-HETE, d_4_-LTB_4_, d_5_-LXA_4_, and d_5_-RvD2), representing each region of the chromatographic analysis to facilitate identification and quantification. Monocyte-derived macrophage incubations were quenched using two volumes of ice-cold methanol containing deuterium-labeled internal standards (500 pg for d_4_-PGE_2_, d_8_–5-HETE, d_4_-LTB_4_, d_5_-LXA_4_, and d_5_-RvD2; 250 pg for d_5_-MaR1, d_5_-MaR2, and d_5_-RvD3; 100 pg for d_5_-RvE1, and 25 pg for d_5_-17R-RvD1). Samples were then extracted using solid-phase extraction columns as in (*37*). Before sample extraction, these were kept at −20°C for a minimum of 45 min to allow protein precipitation. Supernatants were subjected to solid-phase extraction, and methyl formate fractions were collected, brought to dryness, and suspended in phase (methanol/water, 1:1, v/v) for injection on a Shimadzu LC-20 AD HPLC and a Shimadzu SIL-20 AC autoinjector, paired with a QTrap 6500+ (Sciex). An Agilent Poroshell 120 EC-C18 column (100 mm by 4.6 mm by 2.7 μm) was kept at 50°C, and mediators were eluted using a mobile phase consisting of methanol/water/acetic acid of 20:80:0.01 (v/v/v) that was ramped to 50:50:0.01 (v/v/v) over 0.5 min and then to 80:20:0.01 (v/v/v) from 2 to 11 min, maintained until 14.5 min, and then rapidly ramped to 98:2:0.01 (v/v/v) for the next 0.1 min. This was subsequently maintained at 98:2:0.01 (v/v/v) for 5.4 min, and the flow rate was maintained at 0.5 ml/min. QTrap 6500+ was operated using a multiple reaction monitoring method as in (*37*). Each lipid mediator was identified using established criteria; these included matching retention time of the peak of interest with those of authentic or synthetic standards and a signal to noise ratio ≥ 5. Quantitation was carried out in accordance with published methods that included calculating recoveries of deuterium labeled internal standards and linear calibration curves. Where curves for mediator of interest were not available calibration curves for surrogate molecules with similar physical characteristics were employed (*37*–*39*). Calibration curves were obtained for each mediator using lipid mediator mixtures at 0.78, 1.56, 3.12, 6.25, 12.5, 25, 50, 100, and 200 pg that gave linear calibration curves with *r*^2^ values of 0.98 and 0.99.

### Glycolytic and tricarboxylic acid cycle profiling

Samples were placed in one volume of ice-cold MeOH containing deuterated internal standards (5 μg of ^13^C_2_-citrate, 5 μg of ^13^C_2_-fumarate, and 10 μg of ^13^C_6_-glucose) and homogenized. One volume of ice-cold water was added, followed by the addition of two volumes of chloroform. Samples were then centrifuged for 10 min at 4000*g*. Aqueous phase was collected and brought to dryness under a gentle nitrogen stream using TurboVap LV before suspension in H_2_O for LC-MS/MS profiling.

Extracted samples were analyzed using an LC-MS/MS system. Here, a QTrap 6500+ (Sciex) was equipped with a Shimadzu SIL-20 AC autoinjector and LC-20 AD binary pump (Shimadzu Corp.). Separation was conducted with a method adapted from (*40*). Briefly, a Synergi Hydro-RP column (250 mm by 4.6 mm by 4 μm, Phenomenex), maintained at 30°C, was used with a gradient of methanol/water/acetic acid of 0:100:0.5 (v/v/v) that was ramped to 100:0:0.5 (v/v/v) over 16 min. The flow rate was maintained at 0.5 ml/min. To monitor and quantify the levels of analytes, a multiple reaction monitoring method was used as in (*41*). Table S3 reports the transitions used for each of the molecules. Calibration curves were obtained using a mixture of standards for each of the molecules of interest with an *r*^2^ of 0.98.

### Western blot protocol

Colons were homogenized in ice-cold radioimmunoprecipitation assay (RIPA) lysis buffer (Thermo Fisher Scientific, #89900) containing protease and phosphatase inhibitor cocktails by using a Precellys 24 homogenizer (1× 30 s, 5500 rpm). These were then transferred to Eppendorf tubes and centrifuged (15,000 rpm, 4°C) for 15 min. Protein concentration was determined using the bicinchoninic acid protein assay reagent (Pierce) (Thermo Fisher Scientific, #23225). Protein samples were prepared in Laemmli sample buffer (Bio-Rad, #1610747) and resolved on 4 to 15% Mini-PROTEAN TGX Precast Protein Gels (Bio-Rad, #4561084), electrotransferred to nitrocellulose membrane, and subsequently blocked in 5% nonfat dry milk in tris-buffered saline with 0.1% Tween 20 buffer (TBS-T) for 1 hour at room temperature. The membranes were incubated overnight at 4°C with rabbit anti–COX-2 polyclonal antibody (1:500; Abcam, #Ab15191) and mouse anti–glyceraldehyde-3-phosphate dehydrogenase (GAPDH) monoclonal antibody (1:5000; Abcam, #Ab8245). Membranes were then incubated with secondary antibodies: horseradish peroxidase (HRP)–conjugated goat anti-rabbit or HRP-conjugated goat anti-mouse IgG (1:5000), respectively, for 1 hour at room temperature. Protein expression was visualized using the enhanced chemiluminesence reagents (Bio-Rad, #1705060) and quantified using ImageJ software. Protein expression was normalized against GAPDH signal.

### Protein in-gel digestion and peptide LC-MS/MS analysis

Colons were collected in RIPA buffer, homogenized, and protein lysate–quantified as outlined above, and in-gel digestion was carried out as previously described (*22*). Briefly, 60 μg of protein lysate was loaded onto a NuPAGE 7%, tris-acetate (Invitrogen) with NuPAGE LDS sample buffer 1× (Invitrogen) and 50 mM dithiothreitol (DTT). Gel was stained using 0.05% Coomassie Brilliant Blue R-250 (Sigma-Aldrich).

Bands corresponding to a molecular weight of around 68 kDa were excised from the gel and cut in 1 mm–by–1 mm pieces. Gel pieces were then destained with 200 μl of MS grade methanol:50 mM NH_4_HCO_3_ (1:1, v/v) for 1 min with intermittent vortexing twice and dehydrated for 5 min in 200 μl of MS grade acetonitrile:50 mM NH_4_HCO_3_ (1:1, v/v) with intermittent vortexing. Gel pellets were washed 200 μl of 100% acetonitrile for 1 min and then dried under a stream of nitrogen. Gel pellets were rehydrated in 100 μl of 25 mM DTT in 50 mM NH_4_HCO_3_ [≥99.5% (T); BioUltra, Sigma-Aldrich] and incubated for 20 min at 56°C. Supernatant was discarded, 100 μl of 55 mM iodoacetamide (BioUltra, Sigma-Aldrich) in 50 mM NH_4_HCO_3_ was added, and gel pellets were incubated in the dark for 20 min at room temperature. Gel pellets were dehydrated for 5 min in 200 μl of acetonitrile:50 mM NH_4_HCO_3_ (1:1, v/v) with intermittent vortexing, washed with 200 μl of 100% acetonitrile for 1 min, and then dried under a stream of nitrogen. The gel pellet was rehydrated in 25 μl of protease mixture containing Trypsin Gold (10 ng/μl; Promega) and Glu-C (10 ng/μl; Pierce Glu-C Protease, MS grade, Thermo Fisher Scientific) in 0.01% ProteaseMAX Surfactant (Promega) and 50 mM NH_4_HCO_3_ for 10 min in the presence of stable isotope-labeled internal standard (VGAPFS[L(^13^C6; ^15^N)]K HeavyPeptide AQUA, Thermo Fisher Scientific). The enzyme mixture was then layered with 40 μl of 0.01% ProteaseMAX Surfactant and incubated at 37°C for 2 hours. The digestion was stopped by adding 3 μl of 10% (v/v) formic acid. The supernatant was then collected and centrifuged at 12,000*g* for 10 min at 4°C before injection in LC-MS/MS.

Targeted analysis of the acetylated peptide was performed using a Shimadzu LC-20 AD HPLC and a Shimadzu SIL-20 AC autoinjector, paired with a QTrap 6500+ (Sciex) under positive ionization. Presence of the acetylated and isotope-labeled standards was evaluated using multiple reaction monitoring targeting the following ion pairs 438 > 634 and 413 > 598, respectively, with quadrupole resolution set to “unit.” The identity of each peptide was confirmed by evaluating the MS/MS fragmentation profile generated in the enhance product ion mode, which was coupled with the multiple reaction monitoring experiment using information-dependent acquisition. An Agilent Poroshell 120 EC-C18 column (100 mm by 4.6 mm by 2.7 μm) was used to elute the peptides. This was kept at 50°C, and peptides were eluted using a mobile phase consisting of methanol/water/acetic acid of 10:90:0.01 (v/v/v) that was maintained for 0.3 min and then ramped to 70:30:0.01 (v/v/v) over the subsequent from 0.3 to 15 min; this was then ramped to 90:10:0.01 (v/v/v) over the subsequent 2 min and maintained for the remaining 3 min. The flow rate was maintained at 0.2 ml/min for the duration of the experiment. The instrument parameters used are reported in table S4. Synthetic VGAPFS(Ace)LK, corresponding to the peptide fragment containing the acetylated COX-2 serine-516, was obtained from Biomatik and used as a reference standard for the identification of the endogenous acetylated peptide.

### Statistical analyses

We performed all statistical analyses and data derivation using MetaboAnalyst 4.0 (*42*), Prism 8, and Microsoft Excel. Results presented in the figures are expressed as means, and those displayed in the tables are displayed as means ± SEM.

Differences between two groups were determined using one-sample *t* test or Wilcoxon signed-rank test (normalized data), Mann-Whitney test (non-normalized data, two groups). For assessment of more than two groups, we used one-way analysis of variance (ANOVA) and Dunn’s multiple comparisons post hoc test or two-way ANOVA. Sample sizes for each experiment were determined on the variability observed in prior experiments. Partial least squares discriminant analysis (PLS-DA) and orthogonal PLS-DA were performed using MetaboAnalyst 4.0 (*42*) and applying mean centering and unit variance scaling on lipid mediator concentrations. PLS-DA builds a multivariate model that identifies the most relevant variables (lipid mediator concentrations) contributing to the separation of observations (samples) into classes (e.g., aspirin doses). During classification, observations were projected onto their respective cluster. The score plot illustrates the clusters of observations where closer plots represent higher similarity in an observation profile.

## Notes

View/request a protocol for this paper from *Bio-protocol*.
