# Supplementary material for: Aspirin activates resolution pathways to reprogram T cell and macrophage responses in colitis-associated colorectal cancer
Source: Sci Adv. 2022 Feb 2;8(5):eabl5420. doi: 10.1126/sciadv.abl5420 (PMC8809687; doi:10.1126/sciadv.abl5420)
Supplement: Supplementary file 1 — Figs. S1 to S8 Tables S1 to S4 [file sciadv.abl5420_sm.pdf]

Science Advances
AAAS

Supplementary Materials for

Aspirin activates resolution pathways to reprogram T cell and macrophage responses in colitis-associated colorectal cancer

Roberta De Matteis, Magdalena B. Flak, Maria Gonzalez-Nunez, Shani Austin-Williams, Francesco Palmas, Romain A. Colas, Jesmond Dalli*

*Corresponding author. Email: j.dalli@qmul.ac.uk

Published 2 February 2022, *Sci. Adv.* **8**, eabl5420 (2022)
DOI: 10.1126/sciadv.abl5420

This PDF file includes:

Figs. S1 to S8
Tables S1 to S4

Supplemental Figures:

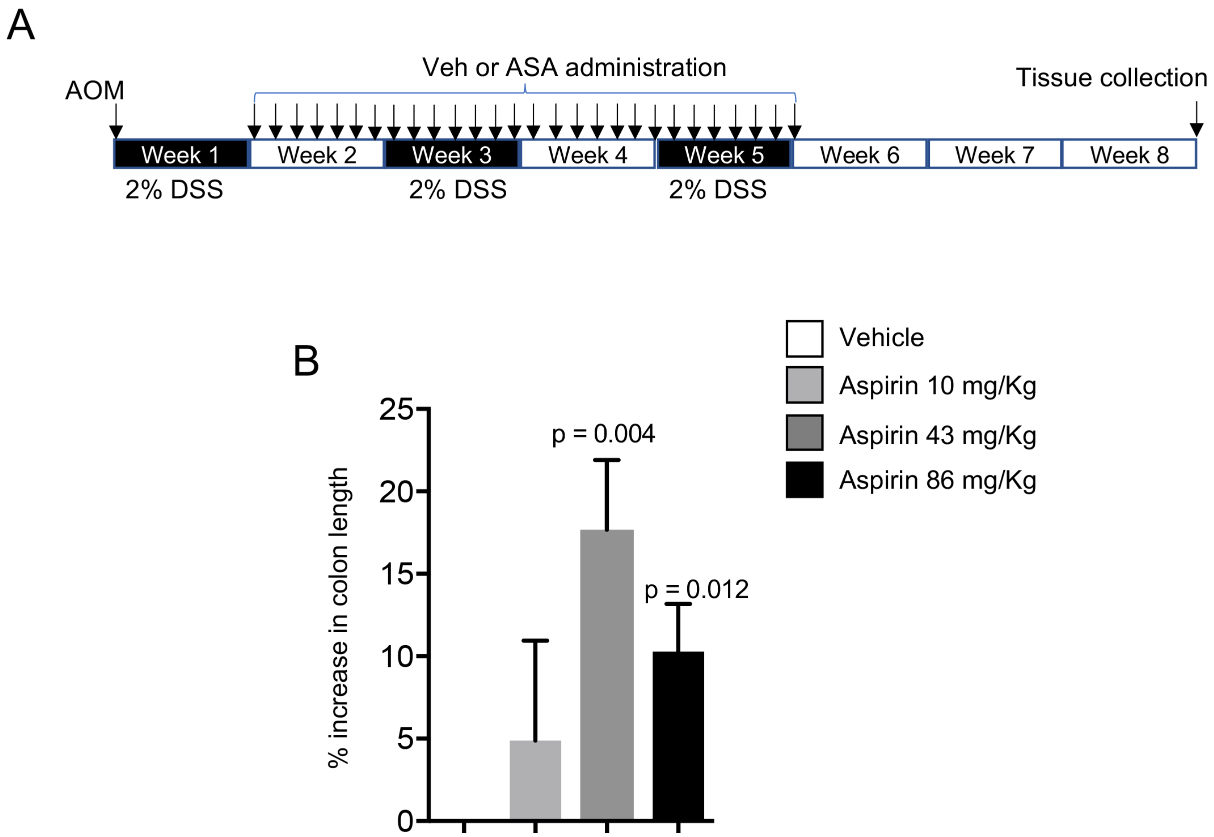

Figure S1: Aspirin protects against colon inflammation in C-CRC. C-CRC was initiated by administrating azoxymethane (7.5mg/Kg via *intraperitoneal* injection) and DSS (2% in the drinking water). After 7 days mice were treated with the indicated doses of aspirin (ASA) or vehicle *via* oral gavage, daily for a four-week period. Three weeks after the last dose of ASA, mice were culled and colons harvested. (A) Experimental timeline. (B) Colon length was determined. Results are mean ± s.e.m. n= 11 mice per group from two distinct experiments. Statistical differences were evaluated using one Sample t-test.

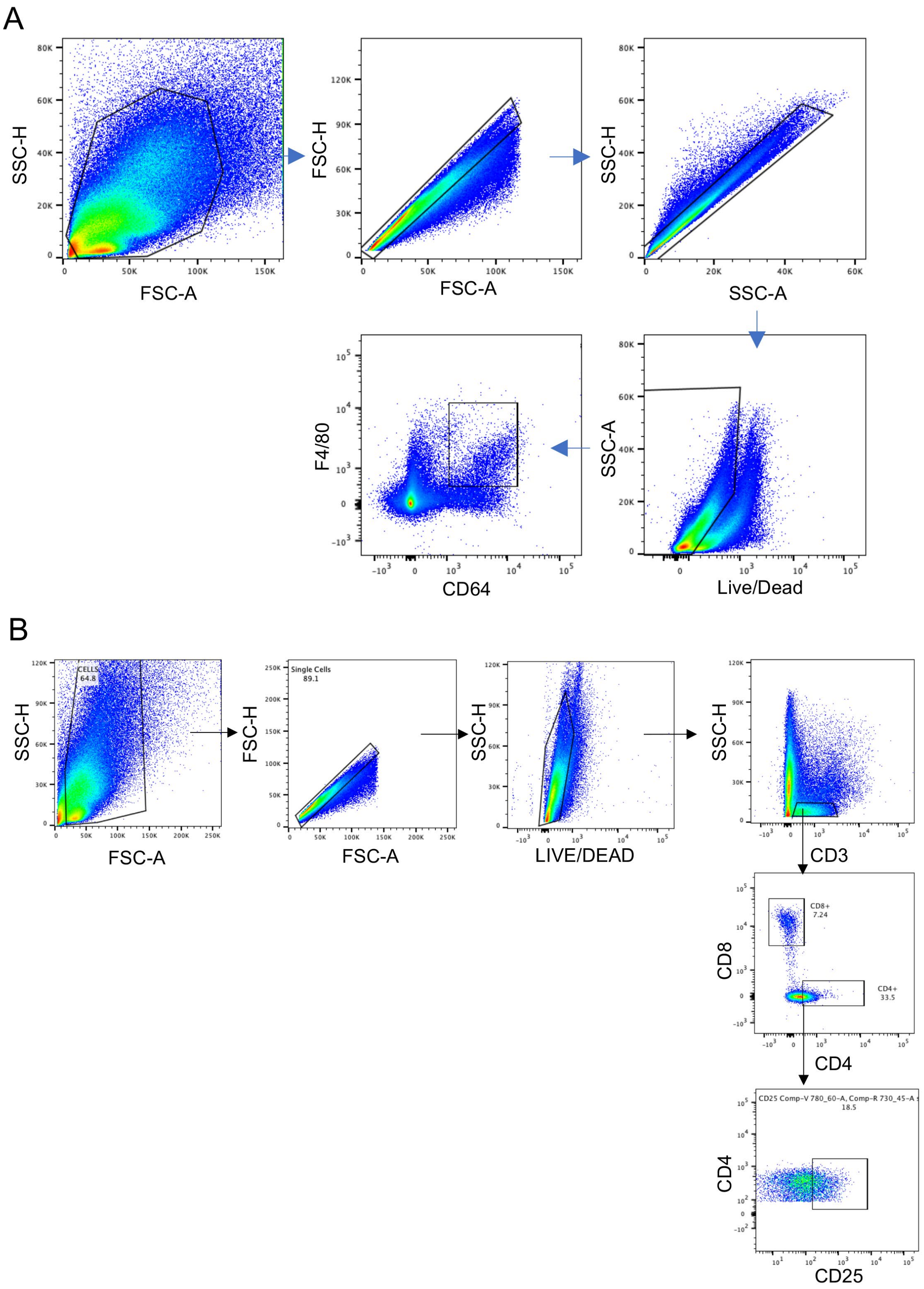

Figure S2: Gating strategies. Strategy employed in the identification of (A) macrophages and (B) T-cell subsets in mouse colonic tissues**.**

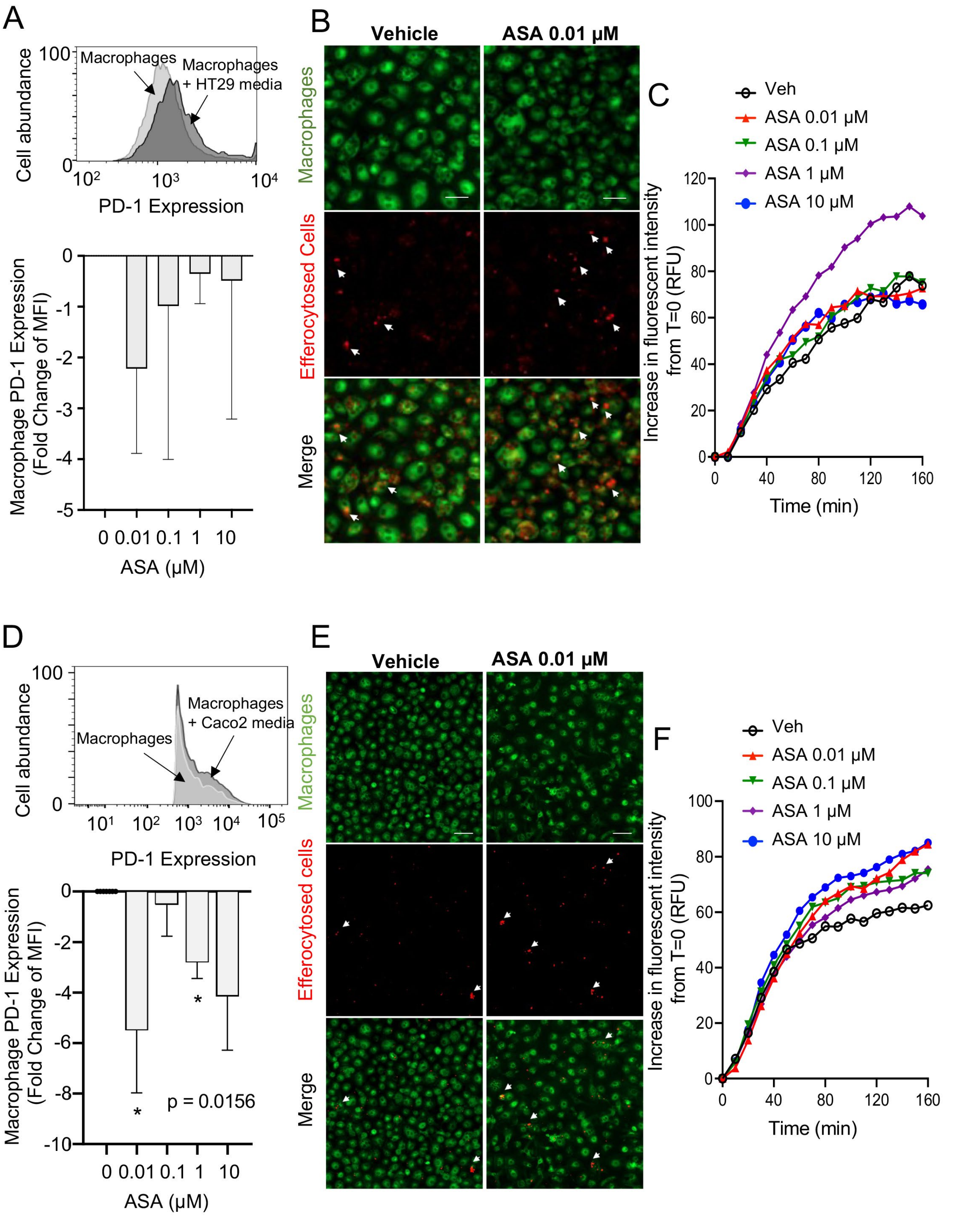

Figure S3: Aspirin decreases PD-1 expression and promotes efferocytosis in macrophages cultured with supernatants from HT-29 and CACO2 colorectal adenocarcinoma cell lines.

(A-D) Human monocyte-derived macrophages were incubated in conditioned media obtained from HT-29 cells. After 8h cells were incubated with the indicated concentrations of aspirin (ASA) or vehicle (PBS containing 0.1% EtOH). Sixteen hours later (A) cells were harvested and PD-1 expression was determined using flow cytometry or (B,C) apoptotic cells were added (at a 5:1 apoptotic cell:macrophage ratio) and efferocytosis monitored for the subsequent 160 minutes using realtime-high content imaging. (B) Representative images, (C) time course of macrophage efferocytosis. Results are representative of n=3-4 donors for A and n=4 determinations for B,C. (D-F) Human monocyte-derived macrophages were incubated with conditioned media obtained from CACO-2 cells. After 8h cells were incubated with the indicated concentrations of ASA or vehicle (PBS containing 0.1% EtOH). After 16h (D) cells were harvested and PD-1 expression was determined using flow cytometry or (E, F) apoptotic cells were added (at a 5:1 apoptotic cell:macrophage ratio) and efferocytosis monitored for the subsequent 160 minutes using realtime-high content imaging. (E) Representative images, (F) time course of macrophage efferocytosis. Results are representative of n=9 donors for E, n= 7 donors for F and n=7 donors for E,F. For A,D statistical differences were evaluated using Wilcoxon signed-rank test.

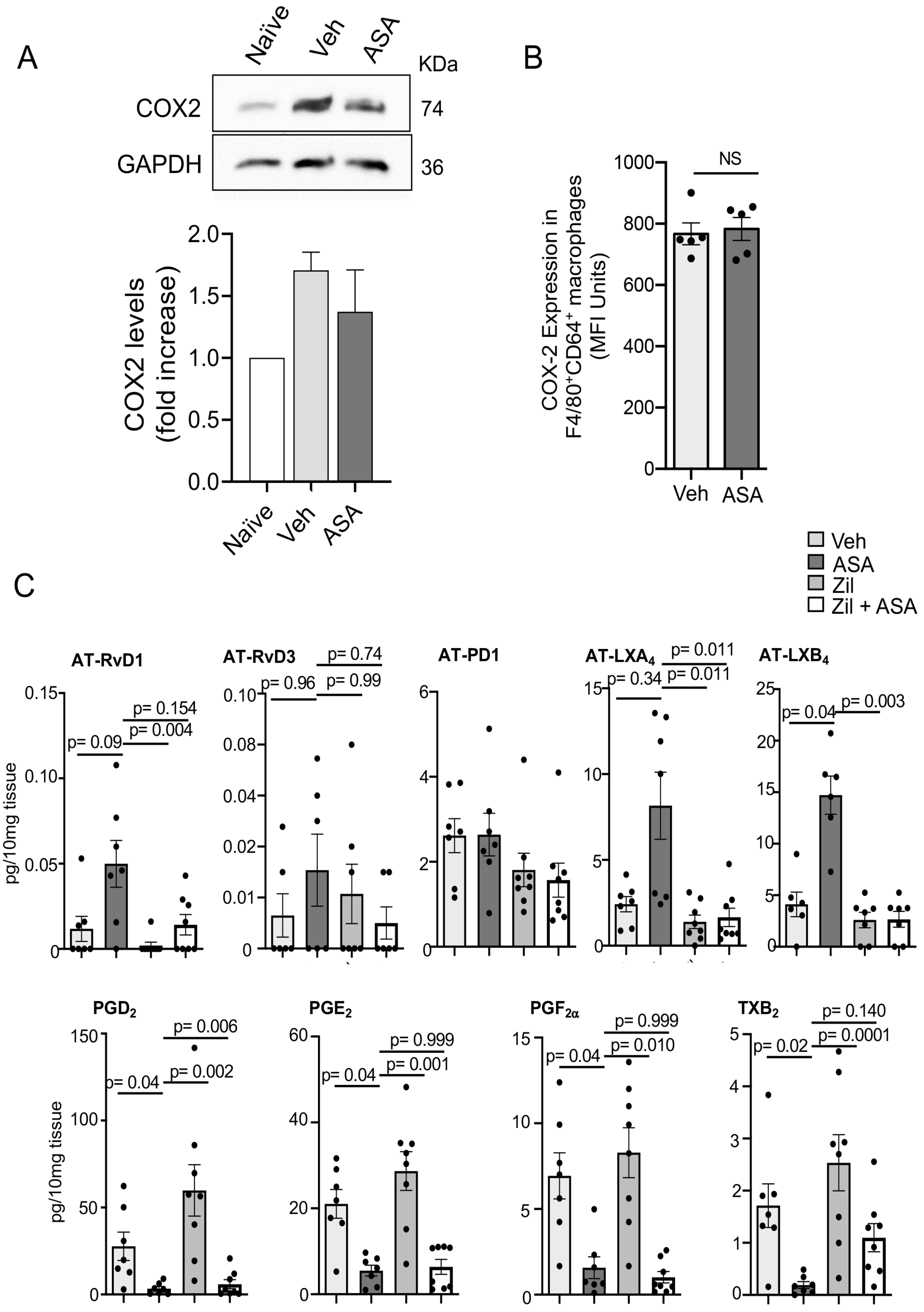
A
Naïve
Veh
ASA
KDa
COX2
74
GAPDH
36
COX2 levels (fold increase)
B
COX-2 Expression in F4/80+CD64+ macrophages (MFI Units)
NS
Veh
ASA
C
Veh
ASA
Zil
Zil + ASA
AT-RvD1
p= 0.09
p= 0.154
p= 0.004
pg/10mg tissue
AT-RvD3
p= 0.96
p= 0.74
p= 0.99
AT-PD1
AT-LXA4
p= 0.34
p= 0.011
p= 0.011
AT-LXB4
p= 0.04
p= 0.003
PGD2
p= 0.04
p= 0.006
p= 0.002
pg/10mg tissue
PGE2
p= 0.04
p= 0.999
p= 0.001
PGF2α
p= 0.04
p= 0.999
p= 0.010
TXB2
p= 0.02
p= 0.140
p= 0.0001

Figure S4: Zileuton inhibits AT-SPM biosynthesis in the colons. (A,B) C-CRC was initiated by administrating azoxymethane (7.5mg/Kg via *intraperitoneal* injection) and DSS (2% in the drinking water). After 7 days mice were treated with 43 mg/Kg of aspirin (ASA) or Vehicle (Veh) *via* oral gavage, daily for an additional week. Tissues were then harvested and the expression of COX-2 (A) in colonic tissues was determined using Western blotting, and (B) in lamina propria macrophages was determined using flow cytometry. Results are mean ± sem. n = 4-5 mice per group. Naieve mice did not receive AOM or DSS. (C) C-CRC was initiated by administrating azoxymethane (7.5mg/Kg *via intraperitoneal* injection) and DSS (2% in the drinking water). After 7 days mice were treated with 43/mg/Kg of aspirin (ASA), zileuton (Zil; 10 mg/Kg), ASA together with Zil or Vehicle (Veh) *via* oral gavage, daily for an additional week, tissues were then harvested and AT-SPM concentrations were assessed using lipid mediator profiling. Results are mean ± sem. n = 6-8 mice per group. Statical differences were evaluated using One-way ANOVA and Dunn's multiple comparison *post hoc* test.

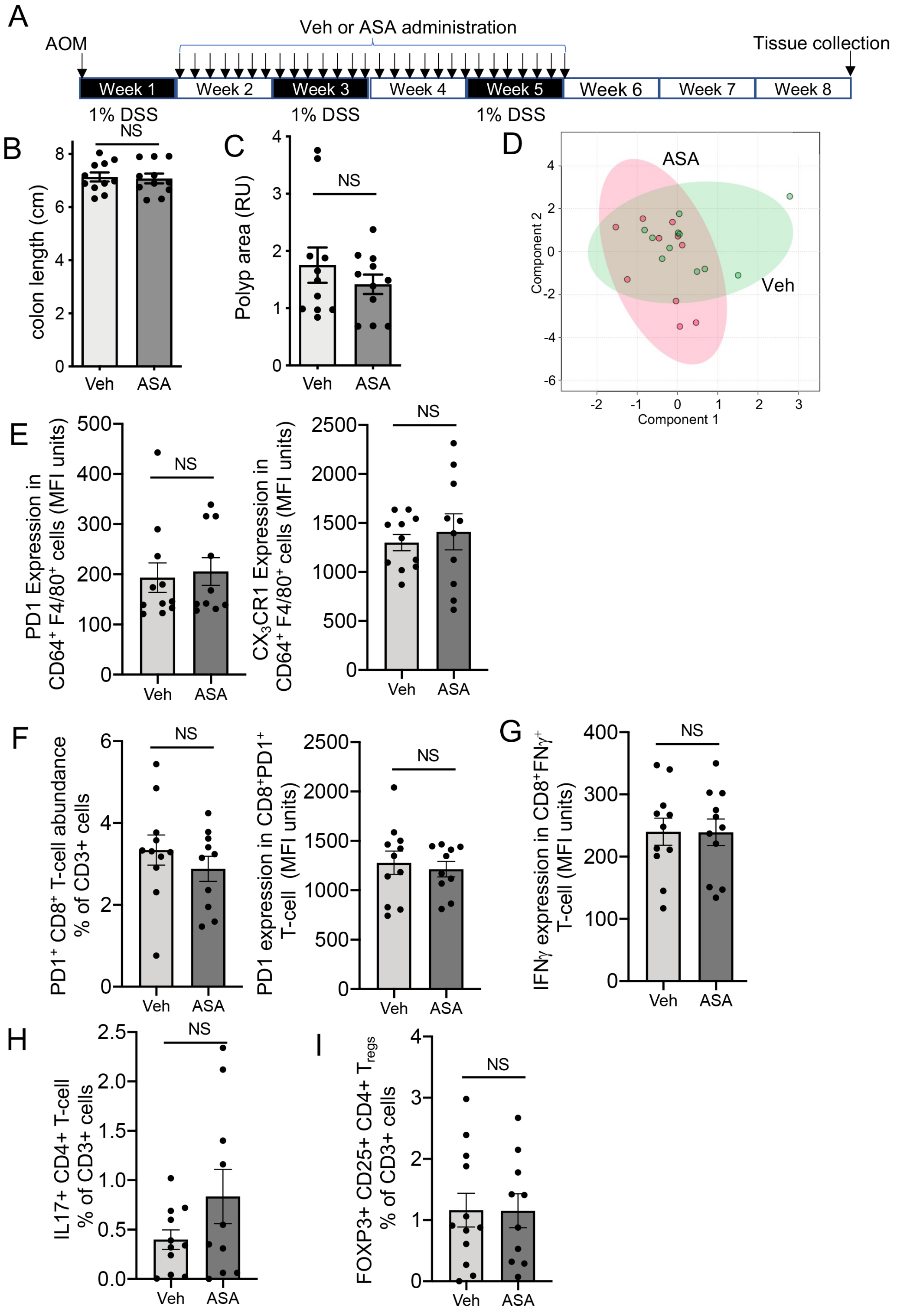

Figure S5: Loss of *Alx/Fpr2* blunts the protective actions of aspirin in C-CRC. C-CRC was initiated by administrating azoxymethane (7.5 mg/Kg via *intraperitoneal* injection) and DSS (1%

in the drinking water). After 7 days mice were treated with aspirin (43mg/Kg; ASA), or Vehicle (Veh) *via* oral gavage, daily for a four-week period. (A) Experimental timeline. (B) Colon length, and (C) polyp area were determined after 7 weeks. Results are mean ± s.e.m. n= 11 mice. (D-I) Lamina propria leukocytes were liberated and (D) Expression of lineage markers was determined using flow cytometry on macrophages and results interrogated using Partial Least Square Discriminant Analysis. (E) Expression of PD-1 and CX_3CR1 was determined on macrophages using flow cytometry. (F) Abundance of PD-1 expressing $CD8^+$ cells (*left panel*) and expression of PD-1 in $CD8^+$ PD-1^+ T-cells (*right panel*). (G) Expression of IFNγ in $CD8^+$ IFNγ^+ cells. (H) Abundance of $CD4^+$IL-$17A^+$ cells and (I) $CD4^+$ $CD25^+$ $FOXP3^+$ T-cells in the colonic lamina propria were determined using flow cytometry. Results are mean ± s.e.m. n = 11 mice per group from two independent experiments.

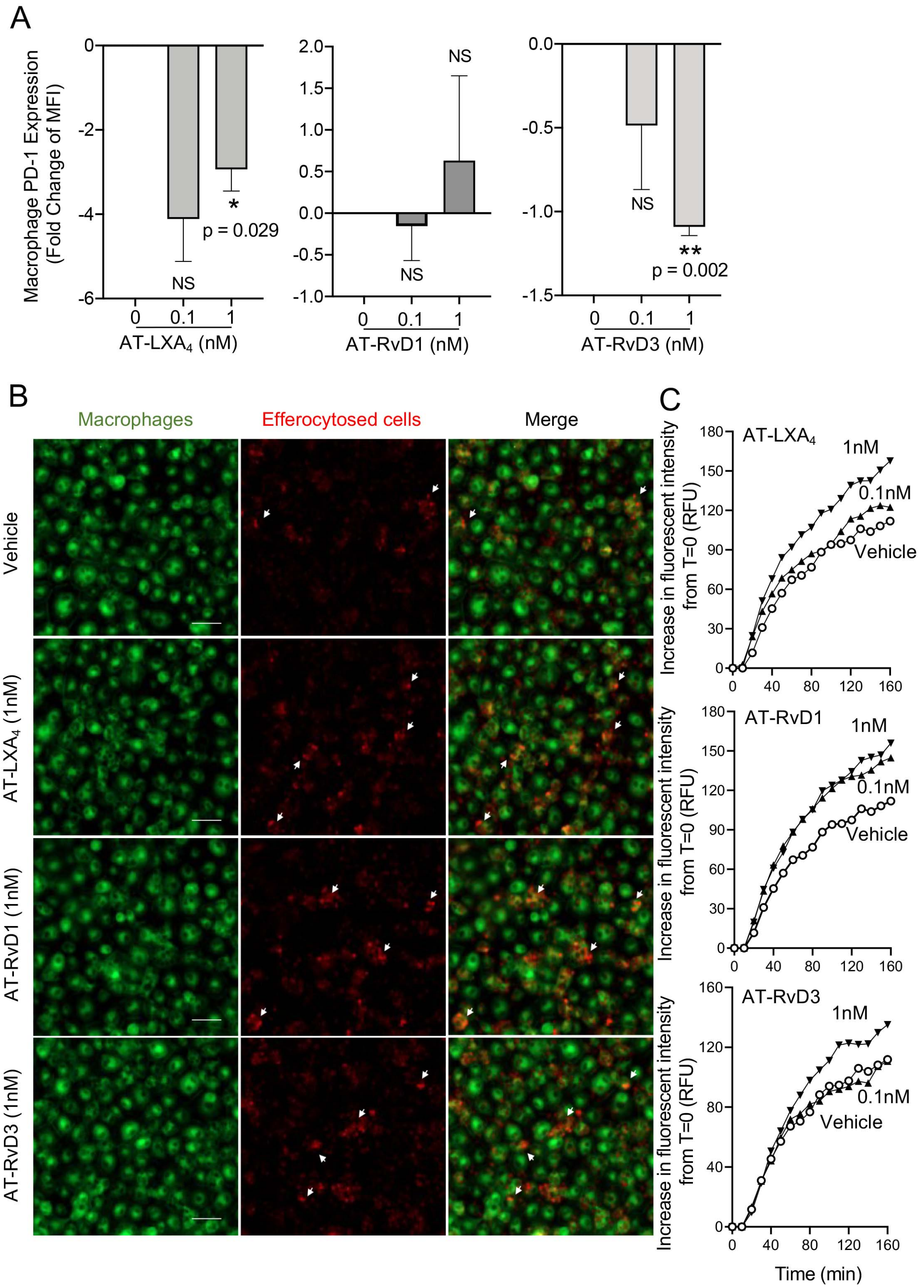

Figure S6: AT-SPM decreases PD-1 expression and upregulates efferocytosis in macrophages cultured with supernatants from HT-29 colorectal adenocarcinoma cell line. Human monocyte-derived macrophages were incubated in conditioned media obtained from HT-

29 cells. After 8h cells were incubated with 0.1 or 1 nM of the indicated AT-SPM or vehicle (PBS containing 0.1% EtOH). Sixteen hours later (A) cells were harvested and PD-1 expression was determined using flow cytometry or (B-C) apoptotic cells were added (at a 5:1 apoptotic cell:macrophage ratio) and efferocytosis monitored for the subsequent 160 minutes using realtime-high content imaging. (B) Representative images, (C) time course of macrophage efferocytosis. Results are mean ± s.e.m of n=3 donors for A and representative of n=4 determinations for B.

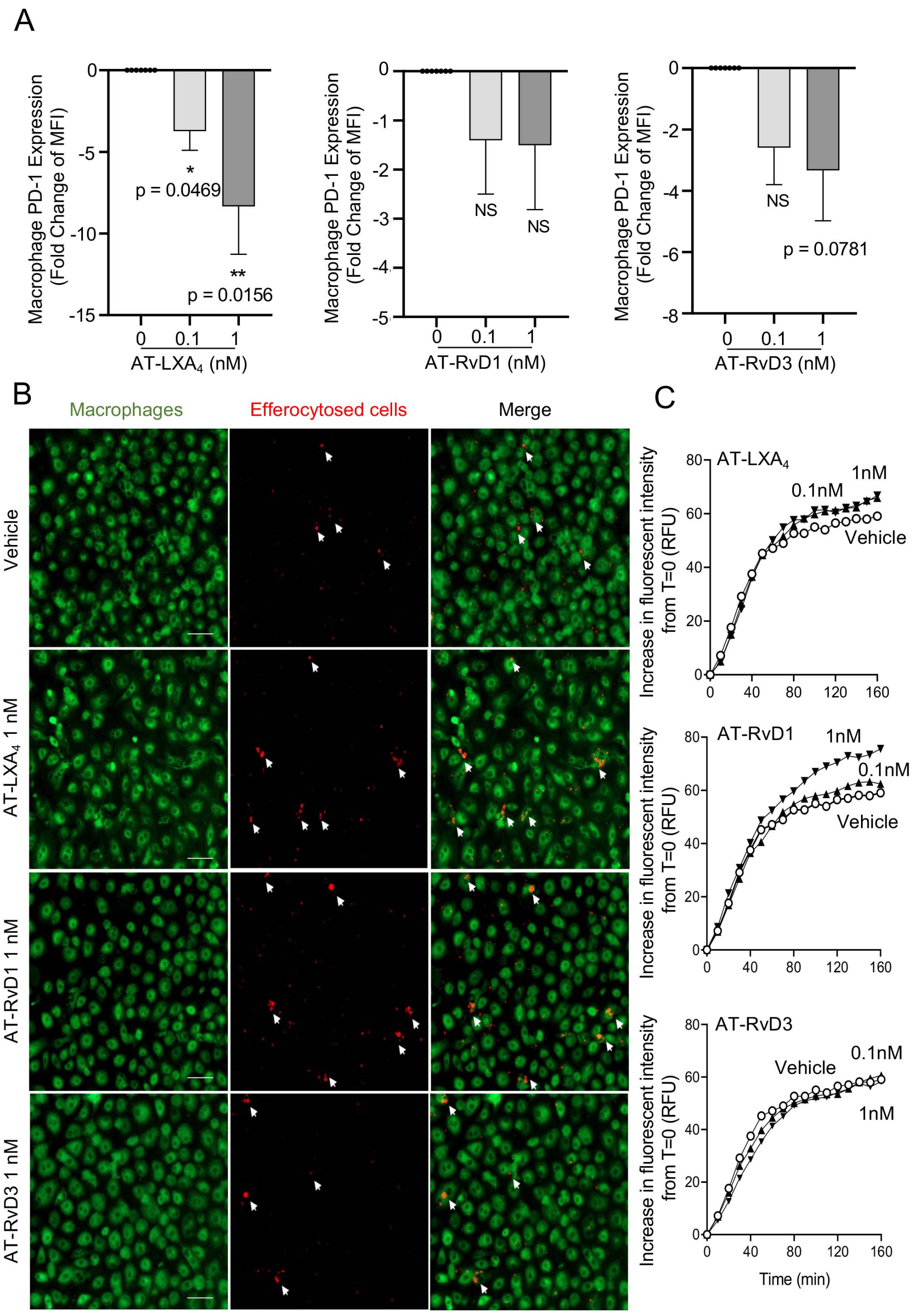

Figure S7: AT-SPM decreases PD-1 expression and upregulates efferocytosis in macrophages cultured with supernatants from CACO-2 colorectal adenocarcinoma cell line. Human monocyte-derived macrophages were incubated with conditioned media obtained

from CACO-2 cells. After 8h cells were incubated with 0.1 or 1 nM of the indicated AT-SPM or vehicle (PBS containing 0.1% EtOH). Sixteen hours later (A) cells were harvested and PD-1 expression was determined using flow cytometry or (B-C) apoptotic cells were added (at a 5:1 apoptotic cell:macrophage ratio) and efferocytosis monitored for the subsequent 160 minutes using realtime-high content imaging. (B) Representative images, (C) time course of macrophage efferocytosis. Results are mean ± s.e.m of n=7 donors for A and representative of n=7 donors and 3 determinations for B and C.

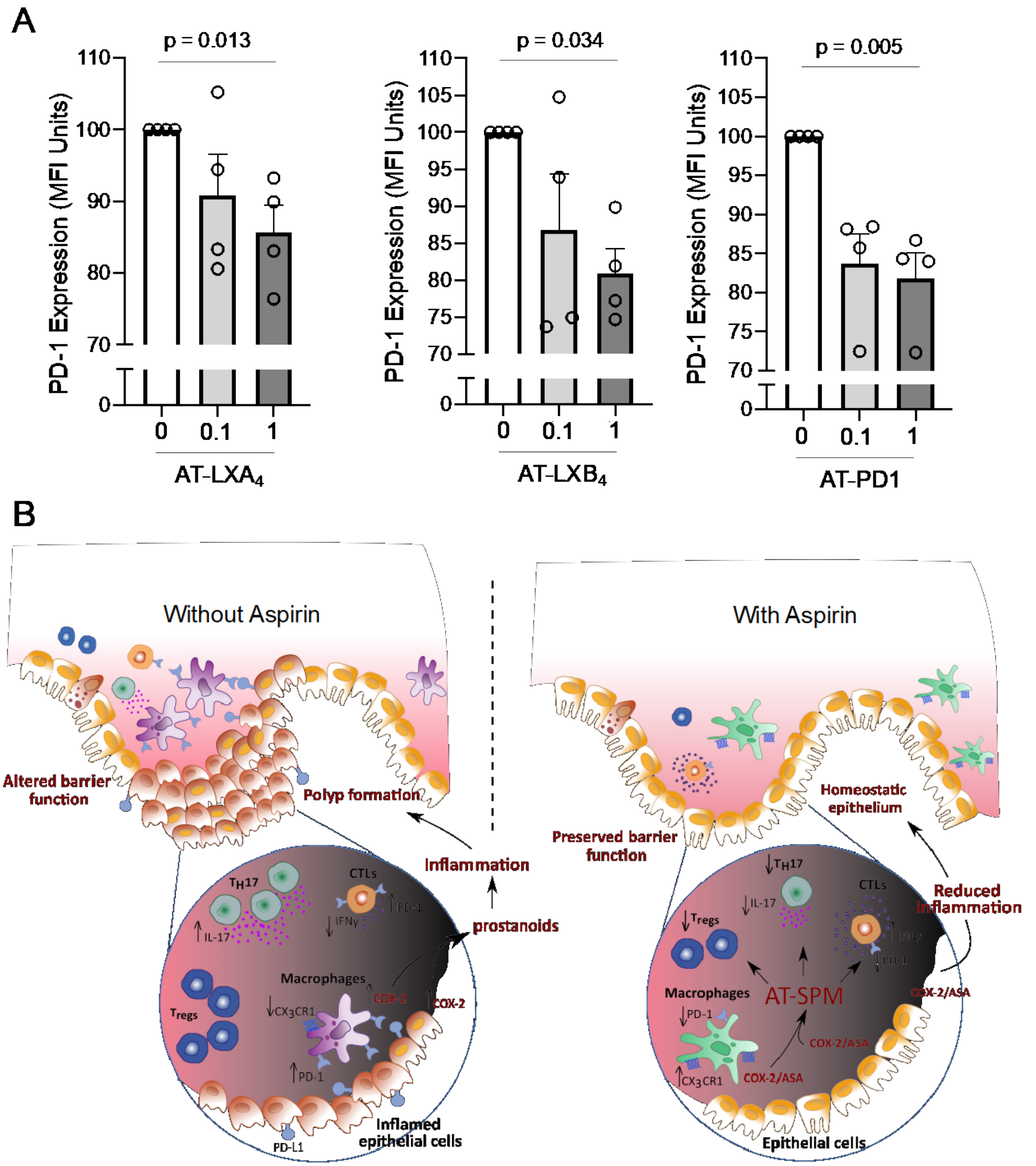

Figure S8: AT-SPM decreases PD-1 expression in CD8+ T-cells. (A) CD8+ T-cells were isolated from peripheral blood mononuclear cells, these were then incubated with CD3/CD28 for 3 days and then with 0.1 or 1 nM of the indicated AT-SPM or vehicle (X-Vivo 15 containing 0.1% EtOH). After 3 days cells were harvested and PD-1 expression assessed using flow cytometry.

Results are expressed as percentage change from Vehicle group and are displayed as mean ± s.e.m. *$P < 0.05$ using one sample t-test. n= 4 volunteers. (B) Illustration summarizing the proposed immune-regulatory actions of aspirin in C-CRC development. Whereby, *via* upregulation of AT-SPM aspirin prevents the upregulation of PD-1 in CD8+ T-cells and macrophages sparing their cytotoxic and pro-resolving actions respectively. Furthermore, AT-SPM upregulation limits the increase in pro-angiogenic Th-17 cells and immunosuppressive T_{regs} in the inflamed lamina propria.

Supplemental Tables:

Table S1: Aspirin regulates SPM formation in human monocyte-derived macrophages

	Transition		Vehicle (pg/x10^6 cells)			Aspirin (pg/x10^6 cells)			Percent Change
**DHA Bioactive Metabolome**	**Q1**	**Q3**	Mean	±	s.e.m.	Mean	±	s.e.m.	
RvD1	***375***	***233***	13.07	±	2.39	10.92	±	1.42	-11.83
RvD2	***375***	***215***	74.68	±	10.28	72.08	±	21.37	-1.83
RvD3	***375***	***137***		-			-		
RvD4	***375***	***101***	75.29	±	6.84	68.07	±	5.99	-7.75
RvD5	***359***	***199***	73.22	±	11.33	67.03	±	14.86	-10.51
RvD6	***359***	***159***	38.73	±	9.61	34.44	±	4.40	-6.19
AT-RvD1	***375***	***233***	21.80	±	1.57	21.40	±	2.11	-2.13
AT-RvD3	***375***	***147***		-			-		
PD1	***359***	***153***	40.63	±	8.27	36.98	±	4.45	-0.58
AT-PD1	***359***	***153***	4.34	±	1.26	4.54	±	1.40	4.67
10S,17S-diHDAH	***359***	***153***	228.13	±	46.94	219.14	±	44.99	-3.70
22-OH-PD1	***375***	***137***		-			-		
MaR1	***359***	***221***	130.61	±	53.65	144.19	±	43.54	23.24
MaR2	***359***	***191***	360.35	±	24.47	348.45	±	38.61	-0.93
22-OH-MaR1	***375***	***221***	321.24	±	37.51	313.15	±	29.28	-0.20
22-COOH-MaR1	***221***	***221***		-			-		
14-oxo-MaR1	***357***	***248***		-			-		
7S,14S-diHDHA	***359***	***177***	56.60	±	14.47	59.74	±	11.54	19.86
4,14-diHDHA	***359***	***159***	117.10	±	18.81	104.52	±	13.92	-3.98
**n-3 DPA Bioactive Metabolome**									
RvT1	***377***	***193***	23.90	±	3.75	23.03	±	3.79	-3.11
RvT2	***377***	***197***	1.63	±	0.65	1.57	±	0.65	UC
RvT3	***377***	***197***		-			-		
RvT4	***361***	***211***	42.93	±	21.15	43.22	±	10.82	40.99
$RvD1_{n\text{-}3\ DPA}$	***377***	***143***	7.51	±	1.03	6.80	±	1.83	-6.23
$RvD2_{n\text{-}3DPA}$	***377***	***233***		-			-		
$RvD5_{n\text{-}3DPA}$	***361***	***143***	152.42	±	27.74	177.40	±	21.93	35.18
$PD1_{n\text{-}3\ DPA}$	***361***	***155***	21.74	±	9.79	24.18	±	9.71	21.41
$PD2_{n\text{-}3\ DPA}$	***361***	***233***	32.54	±	14.44	38.89	±	14.41	32.58
10S, 17S-diHDPA	***361***	***183***	4.52	±	3.33	4.17	±	1.88	0.00
22-OH-$PD1_{n\text{-}3\ DPA}$		***183***		-			-		
$MaR1_{n\text{-}3\ DPA}$	***361***	***223***		-			-		
$MaR2_{n\text{-}3\ DPA}$	***361***	***193***	49.32	±	16.46	49.00	±	13.90	7.89
7S,14S-diHDPA	***361***	***223***	24.85	±	6.94	19.03	±	2.20	-16.34
**EPA Bioactive Metabolome**									
RvE1	***349***	***195***		-			-		
RvE2	***333***	***199***	214.77	±	32.94	192.83	±	25.49	-9.37
RvE3	***333***	***201***	60.61	±	8.75	65.51	±	6.07	16.31
**AA Bioactive Metabolome**									
LXA_4	***351***	***115***	548.80	±	81.82	548.33	±	22.52	4.04
LXB_4	***351***	***221***	271.02	±	4.30	293.53	±	20.50	8.20
5S,15S-diHETE	***335***	***115***	179.81	±	11.30	147.87	±	21.12	-18.43
AT-LXA_4	***351***	***217***	414.00	±	31.10	403.02	±	46.98	-1.15
AT-LXB_4	***351***	***115***	9371.00	±	1399.45	9439.36	±	597.10	4.49
13,14-dehydro-15-oxo-LXA_4	***351***	***217***	31.70	±	2.60	27.56	±	4.79	-13.10
15-oxo-LXA_4	***349***	***233***	20.59	±	4.53	23.84	±	8.46	11.18

Human monocytes were incubated with 1nM ASA and differentiated to macrophages. Lipid mediators were then identified and quantified using lipid mediator profiling. Results are mean ± s.e.m. n = 4 healthy volunteers. - = below limits of the assay. UC = Unchanged

Table S2: Antibodies for Flow Cytometry and ImageStream

Antibody	Clone	Manufacturer	Cat No
PE/Cy5 anti-mouse/human CD11b	M1/70	Biolegend	101210
Brilliant Violet 650™ anti-mouse I-A/I-E	M5/114.15.2	Biolegend	107641
APC/Cyanine7 anti-mouse F4/80	BM8	Biolegend	123118
PerCP-eFluor 710 anti-mouse TIM-4	54 (RMT4-54)	eBioscience™	46-5866-82
Brilliant Violet 711™ anti-mouse CX3CR1	SA011F11	Biolegend	149031
Alexa Fluor® 700 anti-mouse Ly-6G	1A8	Biolegend	127622
PE/Cy7 anti-mouse CD64 (FcγRI)	X54-5/7.1	Biolegend	139314
PE/Dazzle™ 594 anti-mouse IL-10	JES5-16E3	Biolegend	505034
Brilliant Violet 421™ anti-mouse CD279 (PD-1)	29F.1A12	Biolegend	135218
Brilliant Violet 711™ anti-human CD279 (PD-1)	NAT105	Biolegend	367428
Brilliant Violet 421™ anti-mouse LAP (TGF-β1)	TW7-16B4	Biolegend	141408
PE Human/Mouse Arginase 1/ARG1	Polyclonal	R&D	IC5868P
Alexa Fluor® 647 iNOS Antibody	Polyclonal	Novus BIologicals	NBP2-30015AF647
Alexa Fluor® 488 COX-2	Polyclonal	Cell Signaling	13596S
APC/Cyanine7 anti-mouse CD3	17A2	Biolegend	100222
Alexa Fluor® 700 anti-mouse CD4	GK1.5	Biolegend	100430
Brilliant Violet 785™ anti-mouse CD25	PC61	Biolegend	102051
FITC anti-mouse CD127 (IL-7Rα)	A7R34	Biolegend	135008
PerCP/Cyanine5.5 anti-mouse CD69	H1.2F3	Biolegend	104522
Brilliant Violet 711™ anti-mouse CD8a	53-6.7	Biolegend	100748
PerCP anti-mouse CD8a	53-6.7	Biolegend	100732
APC/Cyanine7 anti-human CD8	SK1	Biolegend	344714

PE ROR gamma (t)	B2D	eBioscience™	12-6981-82
PE/Cy7 anti-T-bet	4B10	Biolegend	644824
Brilliant Violet 421™ anti-mouse FOXP3	MF-14	Biolegend	126419
Brilliant Violet 650™ anti-mouse IL-17A	TC11-18H10.1	Biolegend	506930
APC anti-mouse IFN-γ	XMG1.2	Biolegend	505810
PE anti-mouse CD152	UC10-4B9	Biolegend	106306
DyLight 405 5-Lipoxygenase	Polyclonal	Novus Biologicals	NB110-58748V
Zombie Aqua™ Fixable Viability Kit	-	Biolegend	423102

Table S3: MRM employed in the quantitation of glycolytic and TCA metabolites.

Compound	Q1	Q3
$^{13}C_6$-Glucose	185.2	92.1
Glucose	179.1	89.1
Fructose	179.1	89.1
Glucose-6P	259.2	97.1
Fuctose-6P	259.2	97.1
Fructose1,6bisP	339.1	97.1
DiOHacetone-P	169.1	97.1
Glyceraldehyde-3P	169.1	97.1
3P Glycerate	185.1	79.1
PenolPyruvate	167.1	79.1
$^{13}C_2$-Citrate	193.2	112.1
$^{13}C_2$-Fumarate	117.2	73.1
a-keto glutarate	145.0	101.1
Citrate	191.0	111.1
Iso-citrate	191.0	111.1
Succinate	117.2	73.3
Malate	133.1	115.4
Cis-aconitate	173.1	85.1
Pyruvate	87.1	87.1
Oxaloacetate	131.1	87.0
Fumarate	115.1	71.1
Lactate	89.2	43.0

Table S4: Mass spectrometer settings for Multiple Reaction Monitoring and Enhance Product Ion Scan employed in the monitoring of acetylated COX-2 -derived peptide.

Parameter	MRM	EPI
Curtain Gas	30	30
Ion Spray Voltage	-4500	-4500
Temperature	440 °C	440 °C
Ion Source Gas 1	45	45
Ion Source Gas 2	70	70
Delustering Potential	80	40
Entrance Potential	10	10
Collision Energy	15	22
Collision Cell Exit Potential	15	-
